# Impairment of α-tubulin and F-actin interactions of GJB3 induces aneuploidy in urothelial cells and promotes bladder cancer cell invasion

**DOI:** 10.1186/s11658-024-00609-2

**Published:** 2024-07-02

**Authors:** Junnan Liu, Xue Wang, Wencheng Jiang, Anca Azoitei, Tim Eiseler, Markus Eckstein, Arndt Hartmann, Stephan Stilgenbauer, Mohamed Elati, Meike Hohwieler, Alexander Kleger, Axel John, Felix Wezel, Friedemann Zengerling, Christian Bolenz, Cagatay Günes

**Affiliations:** 1 Department of Urology, Ulm University Hospital, Helmholtzstr. 10, 89081 Ulm, Germany; 2 Department of Internal Medicine I, Ulm University Hospital, Ulm, Germany; 3 Institute of Pathology, Friedrich-Alexander University, Erlangen, Germany; 4 Department of Internal Medicine III, Ulm University, Ulm, Germany; 5 CANTHER, ONCOLille Institute, University of Lille, CNRS, UMR 1277, Inserm U9020, 59045 Lille Cedex, France; 6 Institute of Molecular Oncology and Stem Cell Biology, Ulm University Hospital, Ulm, Germany; 7 Present Address: Department of Urology, Mayo Clinic College of Medicine and Science, Rochester, MN USA; 8 Present Address: Molecular Pharmacology and Experimental Therapeutics, Mayo Clinic College of Medicine and Science, Rochester, MN USA

**Keywords:** Spindle orientation, Chromosomal imbalance, Invadopodia, Metastasis, MIBC

## Abstract

**Background:**

We have previously identified an unsuspected role for GJB3 showing that the deficiency of this connexin protein induces aneuploidy in human and murine cells and accelerates cell transformation as well as tumor formation in xenograft models. The molecular mechanisms by which loss of GJB3 leads to aneuploidy and cancer initiation and progression remain unsolved.

**Methods:**

GJB3 expression levels were determined by RT-qPCR and Western blot. The consequences of GJB3 knockdown on genome instability were assessed by metaphase chromosome counting, multinucleation of cells, by micronuclei formation and by the determination of spindle orientation. Interactions of GJB3 with α-tubulin and F-actin was analyzed by immunoprecipitation and immunocytochemistry. Consequences of GJB3 deficiency on microtubule and actin dynamics were measured by live cell imaging and fluorescence recovery after photobleaching experiments, respectively. Immunohistochemistry was used to determine GJB3 levels on human and murine bladder cancer tissue sections. Bladder cancer in mice was chemically induced by BBN-treatment.

**Results:**

We find that GJB3 is highly expressed in the ureter and bladder epithelium, but it is downregulated in invasive bladder cancer cell lines and during tumor progression in both human and mouse bladder cancer. Downregulation of GJB3 expression leads to aneuploidy and genomic instability in karyotypically stable urothelial cells and experimental modulation of GJB3 levels alters the migration and invasive capacity of bladder cancer cell lines. Importantly, GJB3 interacts both with α-tubulin and F-actin. The impairment of these interactions alters the dynamics of these cytoskeletal components and leads to defective spindle orientation.

**Conclusion:**

We conclude that deregulated microtubule and actin dynamics have an impact on proper chromosome separation and tumor cell invasion and migration. Consequently, these observations indicate a possible role for GJB3 in the onset and spreading of bladder cancer and demonstrate a molecular link between enhanced aneuploidy and invasive capacity cancer cells during tumor cell dissemination.

**Graphical Abstract:**

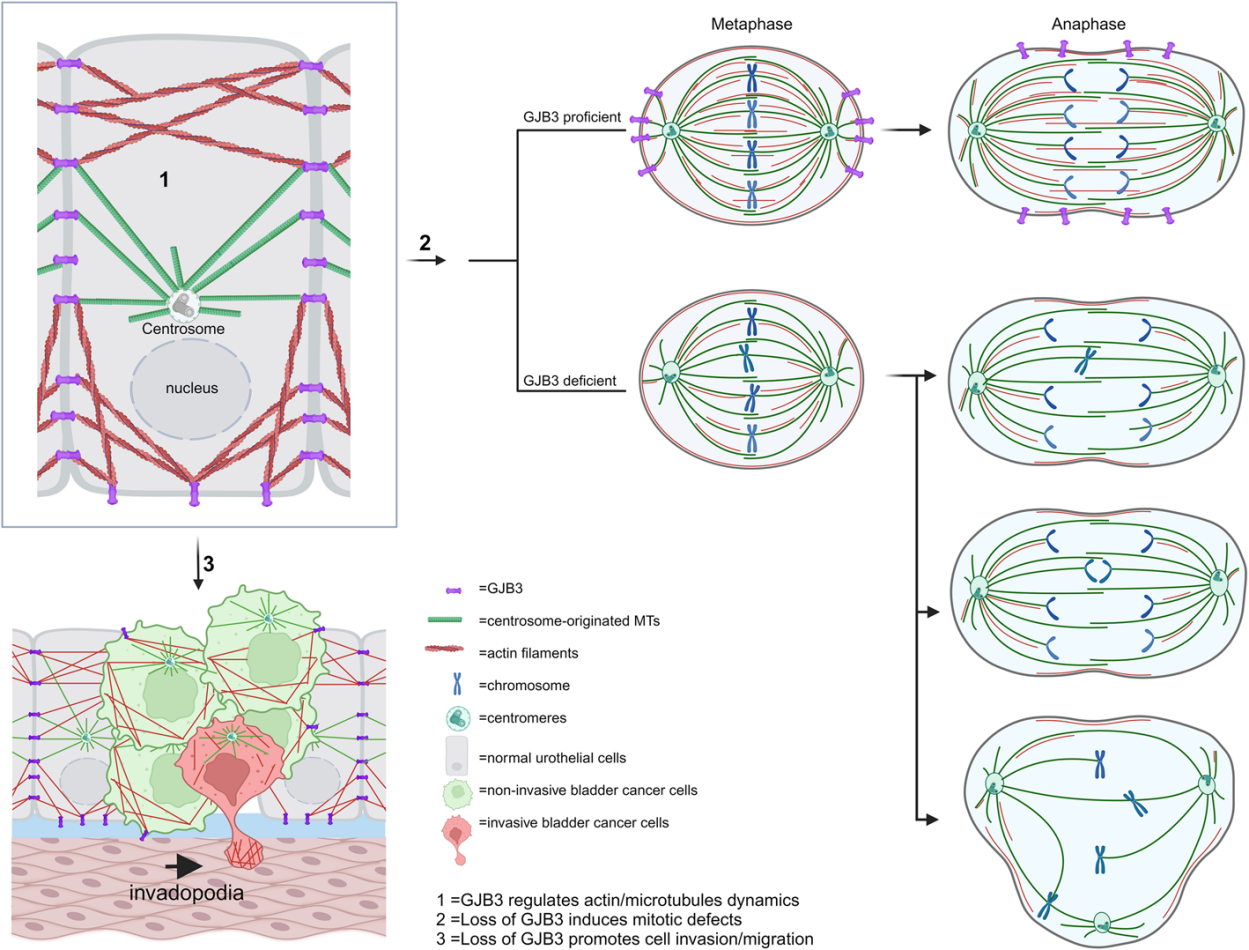

**Supplementary Information:**

The online version contains supplementary material available at 10.1186/s11658-024-00609-2.

## Background

We have previously reported an unsuspected new function of the protein gap junction beta 3 (GJB3), also referred to by Cx31, in ploidy control. GJB3 was shown to be the most promising candidate gene in an RNA interference (RNAi) screening for novel ploidy-control genes. GJB3 knockdown resulted in aneuploidy in multiple cell lines with varying tissue origins, and in mouse hematopoietic stem cells [1, 2]. Furthermore, GJB3 deficiency facilitated tumourigenic transformation of normal human fibroblasts [2]. However, the physiological processes through GJB3 influences cell ploidy and how GJB3 deficiency drives tumorigenesis remain to be elucidated.

GJB3 belongs to the connexin protein family, which are integral in intercellular communication between cells by facilitating the interchange of small molecules that are smaller than 1200 Da, including ions and second messengers [3, 4]. Intercellular channels resulting from gap junctions serve a key role in coordinated tissue response to environmental signals via cell-to-cell communication, also known as the gap junction intercellular communication (GJIC). [5]. Connexins serve numerous cellular processes and many disorders, including carcinogenesis, are influenced by the dysfunction of GJIC [6, 7].

The distribution of gap junction proteins is tissue-specific [8], with corresponding patterns of connexin expression thought to be related to disease pathogenesis. For instance, well-characterized mutations in the connexin gap junction alpha-1 (GJA1, also known as Cx43), which is expressed highly in normal cardiac myocytes and osteoblasts [9], have been shown to result in congenital cardiac abnormalities and impairments in bone development [10, 11]. Similarly, GJB3 is highly expressed in skin, cochlear, and bladder cells [12] and mutations in this protein have been associated with non-syndromic hearing loss in humans and keratinocyte hyperproliferation in transgenic mouse models [13].

At the mechanistic level, connexins can promote cell-to-cell adhesion and extravasation by shaping gap junctions and modulating GJIC, consequently altering motility of cells and their invasive capacity. For instance, GJIC, which are formed by GJA1 improve the adhesion of breast cancer cells to endothelial cells, paving the way for extravasation and metastasis [14]. Besides modulating GJIC, connexins can facilitate the evolution of tumors by influencing multiple cellular mechanisms. For example, GJA1 overexpression impaired the growth of human osteosarcoma cells through increasing the amounts of the CDK (cyclin-dependent kinase)-inhibitor p27, causing and inhibition of G1-S transition in U2OS cells [15]. GJA1 and the protein gap junction beta 2 (GJB2) have been shown to suppress tumor growth by downregulating the fibroblast growth factor receptor-3 [16]. Nevertheless, connexin gene mutations have not been associated with cancer initiation or progression via large-scale genome-wide sequencing data. [17]. On the other hand, an increasing number of reports show that connexin messenger RNA (mRNA) or protein levels may serve as prognostic makers and independently predict patients’ survival. For instance, higher GJB2 or GJA1 protein amounts are associated with a less favorable outcome in breast cancer [18, 19]. Moreover, prognostic prediction of connexins is tissue specific in terms mRNA and protein levels. In this regard, high expression of GJA1 mRNA has been linked to poor survival in glioma tumors [20], whereas high expression of GJB2 mRNA is linked to poor survival in pancreatic cancer [21]. Importantly, loss of the connexion protein Cx31.1, also known as the gap junction beta 5 (GJB5), promotes non-small cell lung cancer, indicating that GJB5 may act as a tumor suppressor protein [22].

The role and significance of connexin proteins have also been investigated in the context of human bladder cancer (BC), and the functions of different connexins in BC can be quite distinct. It was reported that GJB2 contributes to the malignant phenotype in BC through control of intercellular communication [23]. GJA1 expression is elevated in non-muscle invasive BC (NMIBC) samples, and increased GJA1 levels are associated with poor prognosis [24]. Testing for GJA1 levels thus may have utility in the prognostic stratification among patients with high-risk NMIBC. While GBJ3 expression in bladder tissue has been reported [12], virtually nothing is known about the physiological role of GJB3 in bladder tissue or its possible involvement in the onset and dissemination of BC. Given the findings that aneuploidy increases with bladder cancer progression and that the vast majority (up to 90%) of MIBC exhibit a high degree of aneuploidy [25, 26], and the observations showing that DNA ploidy provides predictive characteristics for the survival of patients with BC, it is appealing to investigate the role of GJB3 in BC [27].

Bladder cancer is the tenth most prevalent cancer globally [28], with a higher incidence rate among men in Western Asia, North America, and Europe [29]. While the majority of bladder cancers are non-muscle invasive tumors that can be treated employing bladder preservation strategies, nearly 25% of newly diagnosed patients have muscle invasive disease, which has a high rate for disease progression and cancer-related death. Among patients with non-muscle invasive disease, nearly 20% carry a high-risk disease phenotype characterized by risk for progression to muscle-invasive disease. Although the definitive mechanisms behind progression of bladder cancer remain elusive, aneuploidy, defined as changes in chromosome copy number, is an important genotypic anomaly observed in bladder cancer [30], and is regarded as a potential feature that increases susceptibility for muscle invasion and metastatic progression [30].

The present study aims to explore the molecular mechanisms by which loss of GJB3 results in aneuploidy and GJB3's possible involvement in bladder cancer. Collectively, the results presented in this study identify novel interactions between GJB3 and the cytoskeletal components F-actin and α-tubulin. The presented data indicate that loss of GJB3 impairs proper chromosome segregation through altered microtubule dynamics, revealing a molecular mechanism by which GJB3 downregulation may cause aneuploidy and promote genome instability. On the other hand, the disruption of F-actin-GJB3 interactions alters actin dynamics, which have consequences on the migration and invasion capacities of bladder cancer cell lines. All together, the data indicate a critical role of GJB3 in bladder cancer initiation and progression.

## Methods

### Cell lines

Y235T cells were provided by Dr. Jennifer Southgate (University College London, UK). HBLAK cells were provided by Dr. Michele Hoffmann (Department of Urology, Düsseldorf, Germany). RT4, T24, UMUC3 and UROtsa cells were obtained from Dr. Phillip Erben, University of Heidelberg, Mannheim, Germany). Culturing and experimental manipulation of cells was performed as previously described [31].

### Expression plasmids

The untagged GJB3 overexpression plasmids were cloned in our laboratory (see Table S1 for the cloning primers). Y235T cDNA was used to amplify the open reading frame (ORF) of GJB3 with the Phusion High-Fidelity PCR Kit (ThermoFisher Scientific, Waltham, USA), which was cloned into the EcoRI/SalI (New England Biolabs, Ipswich, UK) digested pBABE-Puro (Robert Weinberg, MIT, Cambridge, MA, USA) or pBABE-Bla (Dr. Hiroshi Nakagawa, University of Pennsylvania) vector. The N-Flag-tagged GJB3 overexpression plasmid was purchased from Genocopia (EX-Q0426, Genecopoeia, MD, USA). The plasmid encoding for the fusion protein End-binding-protein 3 (EB3)-enhanced green fluorescent protein (EB3-eGFP) was provided by Dr Holger Bastians (University of Göttingen, Germany). The EB3-GFP ORF cloning was previously described [31]. LifeAct-Ruby was a gift from Dr. Tim Eiseler (Ulm University Hospital, Ulm, Germany). pLV-MPAct-mCit-IRES-Neo was a gift from Tobias Meyer (Addgene plasmid, #155230; http://n2t.net/addgene:155230; RRID:Addgene_155230).

### Guide RNA and short hairpin RNAs (shRNA) sequences

The control pGIPZ plasmids and the pGIPZ vectors for GJB3 knockdown were purchased from ThermoFisher Scientific (Waltham, USA). The psi-LVRU6H-Hygro lentiviral vectors encoding GJB3-targeting shRNAs and the non-targeting controls were purchased from Genocopia (HSH007486 for GJB3, CSHCTR001 for shScramble; GeneCopoeia, MD, USA). Guide RNAs targeting GJB3 were cloned into the pLentiCRISPR v2 (Addgene vector #52,961) according to the protocol from Zhang Lab (Feng Zheng, Broad Institute, Cambridge, MA, USA). The detailed information for these plasmids is shown in Tables S2 and S3, respectively.

### Total RNA, reverse transcription and quantitative real-time PCR (RT-qPCR)

Commercially available total RNA from apparently healty human tissue samples across different organ types was obtained from Clontech (Total RNA Master Panel II, Cat. No. 636643, Clontech Laboratories, California, USA). Total RNA isolation from cells and tissues, as well as RT-qPCR conditions were conducted exactly as described in Wang et al. [31].

### Antibodies

The following primary antibodies were utilized: Anti-GJB3 rabbit antibody (1:2000 for Western blot (WB) and 1:200 for immunofluorescence (IF), ab236620, Abcam, Cambridge, UK); Anti-GJB3 mouse antibody (1:500 for WB and 1:200 for IHC on mouse samples; 1:400 for immunohistochemistry (IHC) on human samples, sc-81803, Santa Cruz, California, USA); Anti-Flag rabbit antibody (1:2000 for WB, F7425, Sigma-Aldrich, St. Louis, USA); Anti-α-tubulin mouse antibody (1:2000 for WB and 1:500 for IF, T5168, Sigma-Aldrich, St. Louis, USA); Anti-Cortactin mouse antibody (1:500 for IF, #H5, Santa Cruz, California, USA); Anti-γ-tubulin mouse antibody (1:2000 for WB and 1:500 for IF, T5192, Sigma-Aldrich, St. Louis, USA); Anti-β-actin mouse antibody (1:10,000 for WB, A1978, Sigma-Aldrich, St. Louis, USA). Secondary antibodies included: Horseradish peroxidase (HRP) anti-Mouse antibody (1:5000 for IHC, 7076S, Cell Signaling Technology, Danvers, USA); HRP anti-Rabbit (1:2000, 7074S, Cell Signaling Technology, Danvers, USA). One drop of Universal immune-peroxidase anti-Mouse (Nichirei Bioscience, Tokyo, Japan) per piece of tissue was used in IHC. Phalloidin-iFluor647 reagent (1: 1000 for IF, ab176759, Abcam, Cambridge, UK). Anti-Rabbit antibodies were conjugated with Alexa Fluor488 antibody (1:1000 for IF, A11008, ThermoFisher Scientific, Waltham, USA), and anti-Mouse antibodies were conjugated with Cy5 antibody (1:200 for IF, 115–605-006, ThermoFisher Scientific, Waltham, USA).

### Protein extraction and Western blot

Protein extraction and Western blot experiments were performed as described in Wang et al. [31]. Protein samples were loaded onto a 10% acrylamide gel, and subsequently, an immunoblot was conducted in accordance with established procedures [32].

### N-butyl-N-(4-hydroxybutyl)-nitrosamin (BBN)-induced carcinogenesis

The experimental outline for the BBN-induced BC formation in mice was performed as previously described [33]. In summary, 0.1% BBN (Sigma Aldrich Biochemie GmbH, Hamburg, Germany, Cat. No. B8061) was administered to 8-month-old C57BL6 mice in their drinking water ad libitum for a maximum of eight months. Weekly preparations of the drinking water containing BBN were made. After 2, 4, 6, or 8 months of BBN treatment, mice were killed and their bladders collected for histology. The Baden Württemberg Animal Ethics Committee approved the use of animals in the studies (animal experiment number: 35/9185.81-3/1326). Tissue collection and histopathological analyses were performed as described in Wang et al. [31]

### Ureter samples collection and handling

Patient-derived ureter-epithelial cells were prepared from the ureter epithelium of patients undergoing nephrectomy at the University of Ulm following patients’ written consent and Sample treatment and RNA preparation were as described in Wang et al. [31].

### Immunohistochemistry

Hematoxylin and Eosin (H&E) and IHC procedures of human ureter and human/murine BC tissues were carried out, using 4 µm tissue sections by standard staining protocolsas previously described [34]. Additional IHC experiments aimed at assessing GJB3 expression in 243 human muscle-invasive bladder cancer (MIBC) samples and 26 normal human bladder samples were conducted at the University Hospital Erlangen. Signal intensities within stained samples were evaluated by a dedicated genitourinary pathologist (Dr. M. Eckstein) using the Axio Imager A2 microscope (Zeiss, Oberkochen, Germany). The MIBC samples were sourced from a previously described and well-characterized MIBC cohort [35, 36] (also refer to Fig. S2). Ethical approval for this study was granted by the ethical review board of the Friedrich-Alexander-University Erlangen-Nürnberg (Erlangen, Germany; approval numbers 3755 and 329_16B).

### Experimental procedures for the detection of aneuploidy

The protocols for karyotype analysis (chromosome spread assay), identification of lagging chromosomes, and detection of micronuclei and multinucleated cells were the same as in Wang et al. [31]. Additionally, staining with Phalloidin-AF488 was performed to visualize the cell membrane. For the detection of lagging chromosomes, between 270 and 710 cells in mitosis were counted across 3–4 independent experiments. Images were captured using a Zeiss TCS SP5 confocal microscope, utilizing a 63 × objective lens.

### Immunofluorescence

Immunofluorescence was done exactly as described by Wang et al. [31].

### Wound healing and Boyden chamber assays

Cell migration was assessed using both the Wound Healing assay and the Boyden Chamber assay, following the methods outlined in a previous study [32]. In the Wound Healing assay, images were captured using a 5 × objective whereas for the Boyden Chamber assay, pictures were acquired using a 10 × objective. Imaging was conducted with a Zeiss TCS SP5 confocal microscope.

### Determination of spindle orientation

The procedures for determining spindle orientation and calculating the spindle pole displacement factor in response to GJB3 expression levels were essentially identical to those described by Stolz et al. [37].

### Microtubule plus-end tracking with EB3-eGFP

Microtubule plus-end tracking was executed following the methods outlined in a prior study by Becker et al. [38], with modification reported in Wang et al. [31].

### In vitro microtubule-binding assay

Flag-GJB3 was purified from 30 10-cm culture dishes of Y235T-Flag-GJB3 cells by Flag-M2 affinity purification using 500 μl of M2-agarose (Cat.-Nr. A2220, Sigma-Aldrich, St. Louis, USA) according to the manufacturer’s description. Flag-GJB3 was washed 3 times with 200 μl of 100 mm glycine (pH 3.5) in reaction tubes containing 1.5 m Tris (pH 8.8) and the protein was eluted with 150 mM glycine (pH 2.3) in a 15 ml tube with 1 ml Tris (pH 8) buffer to neutralize the acid. Flag-GJB3 was cencentrated using Vivaspin2 and was rebuffed in Tris-buffered saline (TBS). Flag-GJB3 amount and cleanness were determined and binding assays were performed with a microtubule binding protein spin down assay kit according to the manufacturer’s protocol (Cytoskeleton #BK029).

### Ex vivo porcine bladder invasion model

The ex-vivo porcine bladder invasion model was established using methods described previously by Wezel et al. [39]. Images were captured from six randomly selected regions of porcine bladder tissues using a Zeiss TCS SP5 confocal microscope. Magnifications used were 100 × and 200 × , aimed at quantifying the invasive behavior of cells. The distance between cells and the nearest luminal surface of porcine bladder was measured for either the 50 deepest invasive RT4 cells, which gained invasive capacity in response to GJB3 knockdown or the 100 deepest invasive UMUC3 cells, with empty vector or ectopic GJB3. This measurement was performed using the ZEN software with the mean distance was calculated based on these measurements.

### Gelatin degradation assay

For the Gelatin degradation assay, either 3 × 10^4^ RT4 cells (9 × 10^3^ cells per cm^2^) or 1 × 10^4^ UMUC3 cells (3 × 10^3^ cells per cm^2^) were seeded at a density of 20% on Gelatin-FITC coated coverslips in 12-well plates. The RT4 and UMUC3 cells were cultured for 72 or 48 h, respectively. Then, the Gelatin degradation assay was performed as described by Diaz et al. [40].

### Fluorescence recovery after photobleaching (FRAP) imaging of LifeAct-Ruby fluorescence intensity

FRAP experiments were performed as described in Wang et al. [31], according to the protocol described by Weeber et al. [41].

### F-actin co-sedimentation assay

F-actin co-sedimentation assays were conducted following established procedures [42]. Briefly, Flag-GJB3 was purified as described above and purified Flag-GJB3 extracts were 1:10 diluted in F-buffer (consisting of 10 mM imidazole, 75 mM MgCl2, 0.5 mM DTT, 1 mM EGTA, pH 7.2) supplemented with 1% Triton X-100. After centrifugation at 100,000 × g for 20 min at 4 °C to eliminate the lipid-associated protein fraction, the resulting supernatant was collected. Rabbit muscle G-actin (AKL99, Cytoskeleton) was diluted in 500 µl F-buffer and polymerized for 1 h at room temperature (2.5 mg/ml). Next, 25 µl of the purified Flag-GJB3 protein was incubated with 40 µl of F-actin for 30 min at RT. Centrifugation was then carried out for an hour at room temperature at 100,000 × g. To depolymerize the F-actin, the pellet was incubated for one hour in 200 µl of G-buffer (containing 5 mM Tris–HCl, pH 8, 0.5 mM DTT, 0.2 mM CaCl2, and fresh 0.2 mM ATP) after the supernatant was transferred into 1.5 ml tubes. Following this, the pellet and supernatant fractions in equal amounts were analyzed using Western blotting methods.

### Statistical analysis

Statistical analysis and graphical representations were carried out using GraphPad Prism 6 or Excel. A student’s *t*-test was used to compare 2 groups for calculating averages. For standard deviation, the *F*-test was used. Unless otherwise noted, all values are presented as medians with standard errors of the mean (SEM). The Log Rank Test was used to determine overall survival or recurrence-free survival in bladder cancer patients. In all studies, *p*-values < 0.05 were considered statistically significant.

## Results

### GJB3 expression in human tissues

**Fig. 1:**
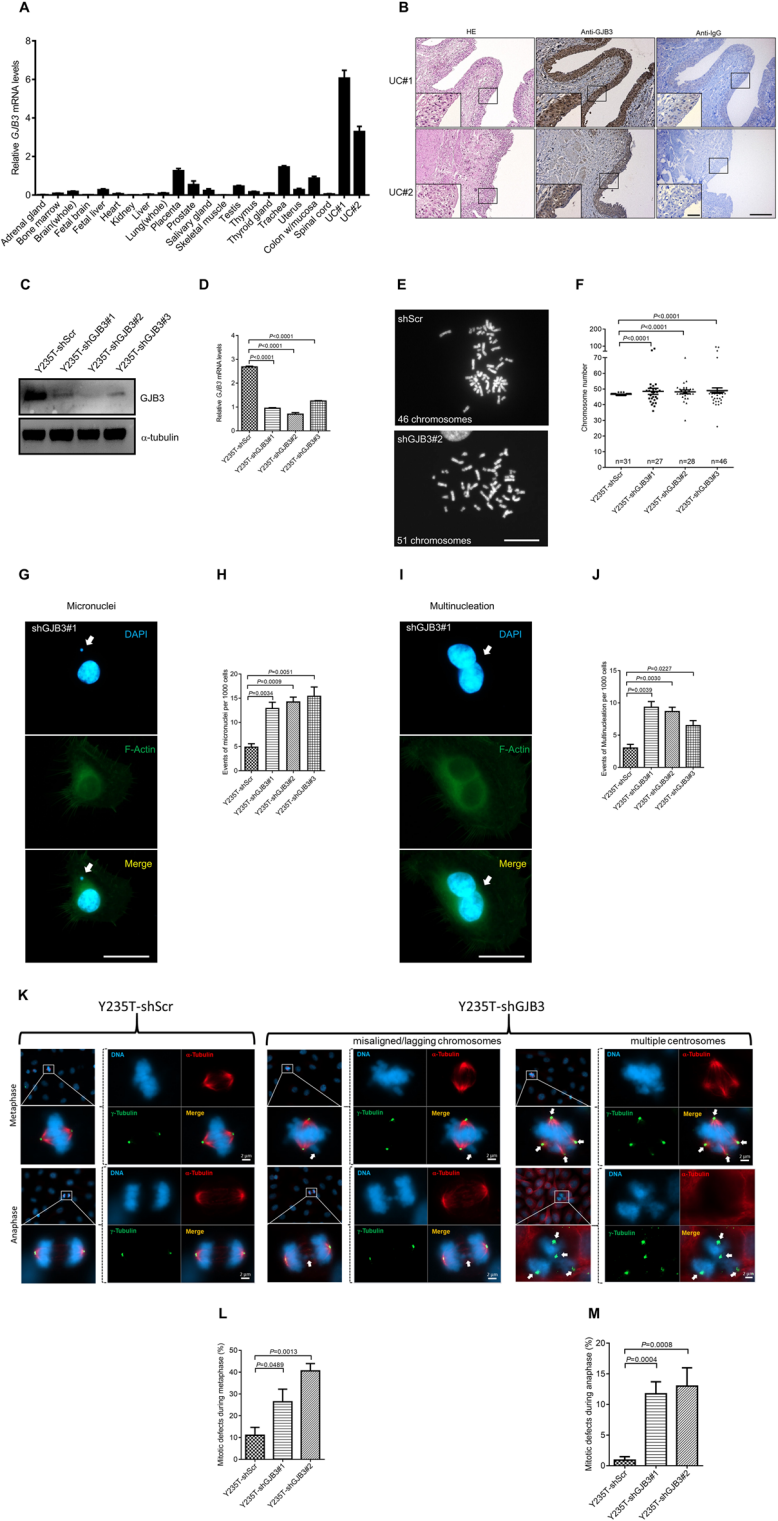
GJB3 controls ploidy in Y235T cells. **A** The presented bar graph illustrates the *GJB3* mRNA amounts across various human tissues, with detailed information available in the Materials and Methods section. Urothelial cells (UC#1 and UC#2) were isolated from ureters from two separate patients who underwent nephrectomy at Ulm University. The mRNA levels were normalized to *GAPDH*. *n* = 3 independent experiments were performed. Error bars represent mean ± SEM.** B** The representative pictures display the HE, GJB3 and IgG staining in human ureter tissues (U#1 and U#2, respectively). **C** The representative Western blot result indicates GJB3 protein levels in Y235T cells with shGJB3. α-tubulin is used as a loading control. *n* = 3 independent experiments were performed. **D** The bar graphs depict the effectiveness of *GJB3* knockdowns at the mRNA level in Y235T cells, with the measurements reference to the *GAPDH* mRNA level. *n* = 3 independent experiments were performed. Error bars represent mean ± SEM. **E** Representative pictures showing metaphase spreads of Y235T cells with shControl and shGJB3#2. Chromosomes are visualized by 4',6-diamidino-2-phenylindole (DAPI) staining. Control cells showing 46 chromosomes in most metaphase spreads. Exemplary pictures demonstrating the induction of aneuploidy in Y235T cells subsequent to GJB3 knockdown. The images show a metaphase spread of Y235T-shGJB3#2 cells with 51 chromosomes. **F** Chromosomes numbers of metaphase spreads from Y235T cells that were knockdown GJB3. *n* = numbers of (Each counting is indicated within the graph). Results are pooled from three independent sets of experiments. Mean ± SEM values are shown in the dot plot, and significance was determined by using Fisher’s exact test.** G** Representative pictures showing micronuclei of Y235T cells with shGJB3#1. Cell nuclei are stained with DAPI, and phalloidin Alexa Fluor 488 was used for F-actin visualization. White arrows indicate micronuclei. **H** Quantitation of cells with micronuclei upon knockdown of GJB3. Results from *n* = 3 separate series of experiments. The bar graph displays the mean ± SEM values, and the two-tailed Student's t-test was used to assess the significance. **I** Immunofluorescence results indicate the multinucleation of Y235T shGJB3#1 cell. Cell nuclei is visualized by DAPI, and F-actin is visualized by Alexa Fluor 488. **J** Quantitation of cells with multinucleation with knockdown of GJB3. Results from *n* = 3 independent sets of experiments. Mean ± SEM values are shown in the bar graph, and the significance was determined by two-tailed Student’s *t*-test. **K** Figures depict of mitotic abnormalities in metaphase and anaphase. DAPI (blue) indicates chromosomes, Cy5 (red) indicates α-tubulin, and Alexa Fluor 488 (green) labeling illustrates γ-tubulin. White arrows are used to indicate chromatid mislocation or multipolar centrosomes. **L**–**M** Quantitative evaluation of mitotic abnormalities. Results from *n* = 3 distinct experiments. The bar graph displays mean ± SEM data, and a two-tailed Student’s *t*-test was used to assess significance. Scale bars: 200 μm (**B** main panels) 50 μm (**B** insets) 20 μm (**E**, **G**, **I**) and 2 μm (**K**). Images are shot at total magnification of 100x (**B** main panels), 630x (**B** insets, **E**, **G**, **I**, **K**)

Messanger RNA levels of *GJB3* were assessed by RT-qPCR in 21 different, apparently healty human tissue samples across different organ types and in ureter-derived tissue epithelium. *GJB3* mRNA levels were found to be highest in ureter epithelium (Fig. 1A). Conversely, among human tissue samples, *GJB3* mRNA levels were lowest in bone marrow, spinal cord adrenal gland, brain, fetal brain, fetal liver, heart, kidney, liver, lung, salivary gland, skeletal muscle, thymus, thyroid gland, and uterus (Fig. 1A). Intermediate levels of GJB3 mRNA expression were observed in human placenta, prostate, testis, trachea, and colonic mucosa. Assessment at the protein-level revealed strong GJB3 expression patterns in epithelial cells of normal human ureter and bladder (Fig. 1B and Fig. S1A) as well as in murine bladder tissue (Fig. S1B). These results imply that GJB3 may have an integral function in the bladder and ureter epithelium in both humans and mice.

### GJB3 knockdown causes cell division defects and aneuploidy in Y235T cells

To investigate the effect of GJB3 on genome stability in the urothelium, three different shRNAs were utilized to downregulate GJB3 gene expression in ureter-derived, telomerase-immortalized Y235T cells. (Fig. 1C, D). GJB3 knockdown in Y235T cells resulted in increased aneuploidy, as measured by metaphase spread analysis (Fig. 1E, F). Chromosome counts varied from 36 to 85 in Y235T-shGJB3#1, from 30 to 70 in Y235T-shGJB3#2, and from 26 to 95 in Y235T-shGJB3#3. As expected, Y235T-shScr cells infected with a control shRNA vector preserved a normal karyotype in the majority of control cells. Please note, some deviation from normal karyotype may occur in control cells as associated with experimental procedures, such as colcemid treatment and loss of few chromosomes during metaphase spreading.

The formation of micronuclei and the appearance of cells with multiple nuclei were evaluated to further assess the impact of GJB3 loss on genome stability. The number of both micronucleated and multinucleated cells was increased in Y235T cells with the GJB3 knockdown (Fig. 1G–J); indeed, reduced GJB3 levels induced a 2.6 to 3.1-fold increase in number of cells with micronuclei (5 events per 1000 cells in shScr versus 13 in shGJB3#1, *P* = 0.0034, 14.3 in shGJB3#2, *P* = 0.0009 and 15.5 in shGJB3#3, *P* = 0.0051; Fig. 1H) and a threefold increase in multinucleated cells (3 events per 1000 cells in shScr versus 9.3 in shGJB3#1, *P* = 0.0039, 8.7 in shGJB3#2, *P* = 0.0030 and 6.5 in shGJB3#3, *P* = 0.0227; Fig. 1J) compared to the control Y235T-shScr cells.

To further elucidate the molecular mechanisms by which GJB3 downregulation induces aneuploidy, the chromosomal architecture of cells with and without GJB3 downregulation was closely analyzed at various mitotic stages (Fig. 1K). Indeed, a 1.4 to 2.7-fold increased number of mitotic defects within Y235T cells was observed in response to GJB3 downregulation, specifically during metaphase (11.0% in shScr versus 26.4% in shGJB3#1, *P* = 0.0489, and 40.6% in shGJB3#2, *P* = 0.0013; Fig. 1L), and 12 to 13-fold increase during anaphase (0.9% in shScr versus 11.7% in shGJB3#1, *P* = 0.0004, and 13.0% in shGJB3#2, *P* = 0.0008; Fig. 1M). Notably, we observed chromosomal segregation and cytokinesis defects associated with multiple centrosomes (Fig. 1K).

### GJB3 regulates spindle orientation and microtubule dynamics

**Fig. 2:**
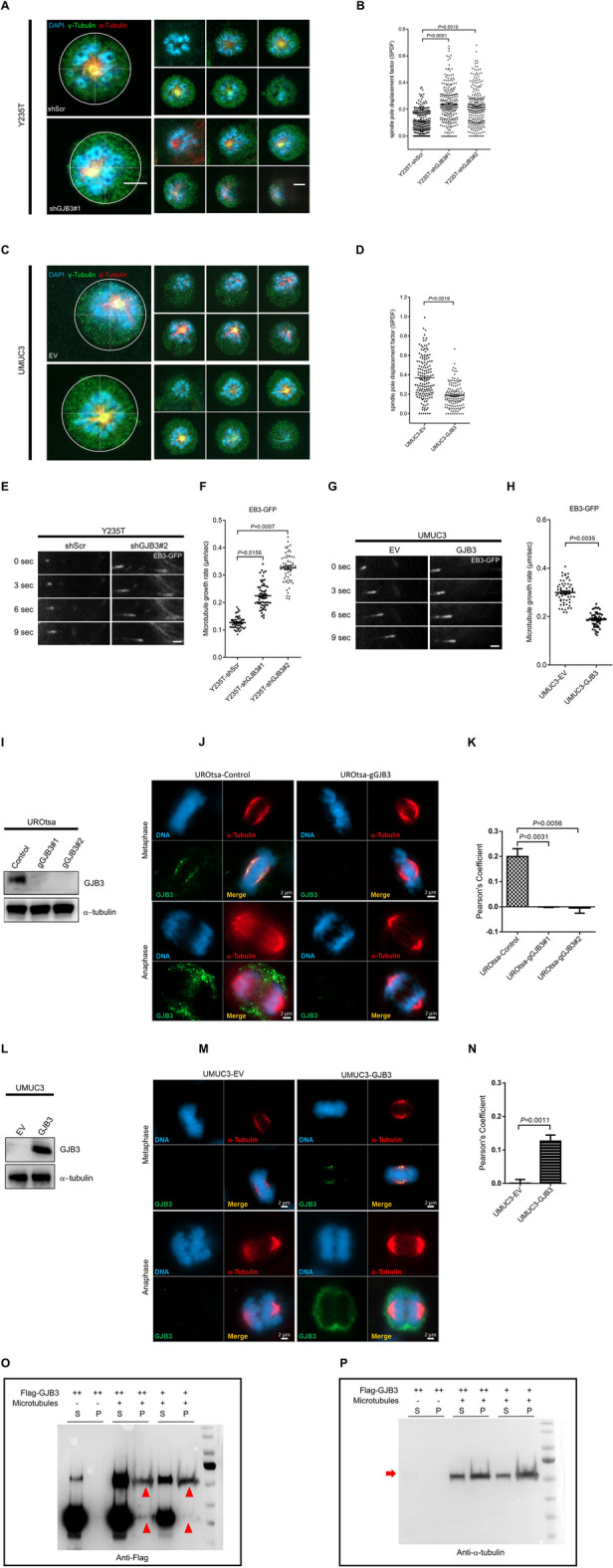
GJB3 controls spindle orientation and microtubule dynamics. **A** Exemplary pictures illustrating the disorientation of Y235T-shGJB3#1 cells. The chromosomes are indicated by DAPI, γ-tubulin is visualized by Alexa Fluor 488, and α-tubulin is visualized by Cy5. **B** Quantitative assessment of the spindle pole displacement factor (SPDF) in Y235T cells. *n* = 256 (Y235T-shScr), 253 (Y235T-shGJB3#1), 263 (Y235T-shGJB3#2), **C**, Representative images showing reorientation of UMUC3 cells with ectopic GJB3. The chromosomes are indicated by DAPI, γ-tubulin is visualized by Alexa Fluor 488, and α-tubulin is visualized by Cy5. **D** Quantitative assessment of the spindle pole displacement factor (SPDF) in UMUC3 cells. *n* = 155 (UMUC3-EV), and 140 (UMUC3-GJB3). Experiments present combined data from three separate sets of independent experiments. The two-tailed Student’s *t*-test was used to evaluate significance, and the mean ± SEM data are displayed in the dot plot. To boost the proportion of prometaphase cells, cells were treated to dimerthylenastron for four hours prior to labeling.** E** Examples of images demonstrating microtubule growth in Y235T cells expressing shGJB3#2. **F** Rates of mitotic microtubule plus-end assembly in Y235T-shGJB3 cells.* n* = 60 cells are pooled from three independent sets of experiments. **G** Example of images demonstrating growth of microtubules in UMUC3-GJB3 cells. **H** Rates of mitotic microtubule plus-end assembly in UMUC3-GJB3 cells.* n* = 60 cells are combined from three separate sets of experiments. The two-tailed Student’s *t*-test was used to evaluate significance, and the mean ± SEM data are displayed in the dot plot. GJB3 interacts with α-tubulin. The deletion of GJB3 in UROtsa cells using the CRISPR-cas9 method was detailed in the main article. **I** Western blot displaying GJB3 protein levels in UROtsa cells with a control guide RNAs directed against green flourescent protein or two distinct gRNAs targeting GJB3 (gGJB3#1 and GJB3#2). α-tubulin serves as loading control. *n* = 3 separate experiments were conducted. **J** Exemplary pictures displaying GJB3 and α-tubulin colocalization in UROtsa cells during metaphase. GJB3 is visualized by Alexa Fluor 488 and α-tubulin is visualized by Cy5. Yellow signal indicates the overlap of GJB3 and α-tubulin. **K** Quantitation of GJB3 and α-tubulin colocalization in UROtsa cells by Pearson’s correlation coefficient. *n* = 49 (UROtsa-gControl), 58 (UROtsa-gGJB3#1), 53(UROtsa-gGJB3#2) are pooled from three to four independent experiments. **L** GJB3 protein level in UMUC3 cells with ectopic GJB3 was detected by Western blot. α-tubulin is used as a loading control. *n* = 3 independent experiments were performed. **M** Representative images displaying the colocalization of GJB3 and α-tubulin in UMUC3 cells during metaphase. GJB3 is visualized by Alexa Fluor 488 and α-tubulin is visualized by Cy5. Yellow signal indicates the overlap of GJB3 and α-tubulin. **N** Quantitation of GJB3 and α-tubulin colocalization in UMUC3 cells by Pearson’s correlation coefficient. *n* = 105 (UMUC3-EV), and 64 (UMUC3-GJB3) are pooled from three to four independent experiments. Mean ± SEM values are shown in the bar graph, and significance was determined by two-tailed Student’s t-test (**M**, **N**). **O**–**P** GJB3 bundle microtubule (MT) filament level was detected by Western blot. 5 × 10^11^ MT/ml and 5–10 μm in length MTs were incubated with increasing concentrations of GJB3 (relative GJB3 amount is indicated by + or + +). Supernatant (S) and pellet (P) were subjected to 10% SDS-PAGE after high-speed centrifugation at 100,000* g*. (**O**), Flag-GJB3, indicated by red arrowheads and (**P**), microtubules, indicated by red arrows, are visualized by specific antibodies. *n* = 3 independent experiments were performed. Scale bars: 5 μm (**A**, **C)** and 1 μm (**E**, **G)** and 2 μm (**J**, **M**). Images were captured at total magnification of 630x

Spindle orientation was examined to evaluate the impact of GJB3 function on mitosis in more detail (Fig. 2A–D). GJB3 knockdown caused mislocation of spindle poles during prometaphase in Y235T cells (Fig. 2A, B), while ectopic expression of GJB3 prevented spindle pole mislocation in UMUC3 cells (Fig. 2C, D). Essentially, GJB3 downregulation in Y235T cells caused a significant increase of spindle pole displacement factor (SPDF) compared to control Y235T cells (0.108 with shScr versus 0.238 with shGJB3#1, *P* = 0.0001, and 0.215 with shGJB3#2, *P* = 0.0010; Fig. 2B). In contrast, ectopic expression of GJB3 in UMUC3 (UMUC3-GJB3) cells results in a 49% reduction of SPDF when compared to UMUC3 control cells with empty vector (UMUC-EV) (0.368 in UMUC3-EV versus 0.187 in UMUC3-GJB3, *P* = 0.0019) (Fig. 2D).

Considering that cancer cells often possess an inherent chromosomal instability related to dysregulation in microtubule dynamics, we sought to assess the impact of GJB3 on microtubule assembly by tracking the plus-end movement of EB3 protein, as described by Ertych et al. [43] (Fig. 2E–H and Supplementary video files). We observed that GJB3 knockdown in Y235T cells significantly increased microtubule assembly rates, as compared to Y235T-shScr cells (GJB3-shScr: 0.126 μm/sec versus 0.224 μm/sec with shGJB3#1, *P* = 0.0156, and 0.327 μm/sec with shGJB3#2, *P* = 0.0007; Fig. 2F). In contrast, ectopic expression in UMUC3 cells reduced the microtubule assembly rates by 37% (0.299 μm/sec in UMUC3-EV versus 0.187 μm/sec in UMUC3-GJB3, *P* = 0.0035) (Fig. 2H).

### GJB3 interacts with α-tubulin during mitosis

The mechanisms underlying the association between GJB3 function and microtubule dynamics during cellular mitosis were further explored based on our observation that GJB3 expression was inversely correlated with microtubule assembly. Colocalization studies were first conducted to understand the interaction between microtubule components and GJB3 protein. To avoid signals from potential endogenous GJB3 expression, CRISPR-Cas9 technology was used to knock-out (KO) GJB3 in UROtsa cells (UROtsa-gGJB3#1, #2) (Fig. 2I), an SV40-LT transformed ureter-derived cell strain with near-normal karyotype [44]. Efforts to generate KO-Y235T cells or other p53-positive KO-cell lines (e.g., the RT4) failed, most likely because endonucleolytic DNA cleavage activity of Cas9 triggered a potent apoptotic response in these cells. UMUC3 cells with and without ectopic GJB3 expression were also utilized for colocalization analyses (Fig. 2L). During metaphase, GJB3 colocalizes with α-tubulin at centrosomes in UROtsa (*r* = 0.1997 in UROtsa-Control versus *r* = − 0.0006 in UROtsa-gGJB3#1, *P* = 0.0031, and *r* = − 0.0046 in UROtsa-gGJB3#2, *P* = 0.0056) as well as in UMUC3 cells (r = 0.0008 in UMUC3-EV versus *r* = 0.1260 in UMUC3-GJB3, *P* = 0.0011)and UMUC3 cells, as shown in immunofluorescence pictures (Fig. 2J, M) and Pearson’s correlation coefficient analysis (Fig. 2K, N). Interestingly, no overlap between GJB3 and α-tubulin was observed during anaphase (Fig. 2J, M).

To evaluate whether GJB3 could interact with microtubule components of, a microtubule binding approach was performed. Flag-GJB3 fusion protein was purified from cell extracts and subjected to in vitro microtubule co-sedimentation approach. The results revealed that GJB3 can also effectively and dose-dependently bind polymerized microtubules (Fig. 2O, P).

### The role of GJB3 in bladder cancer

**Fig. 3:**
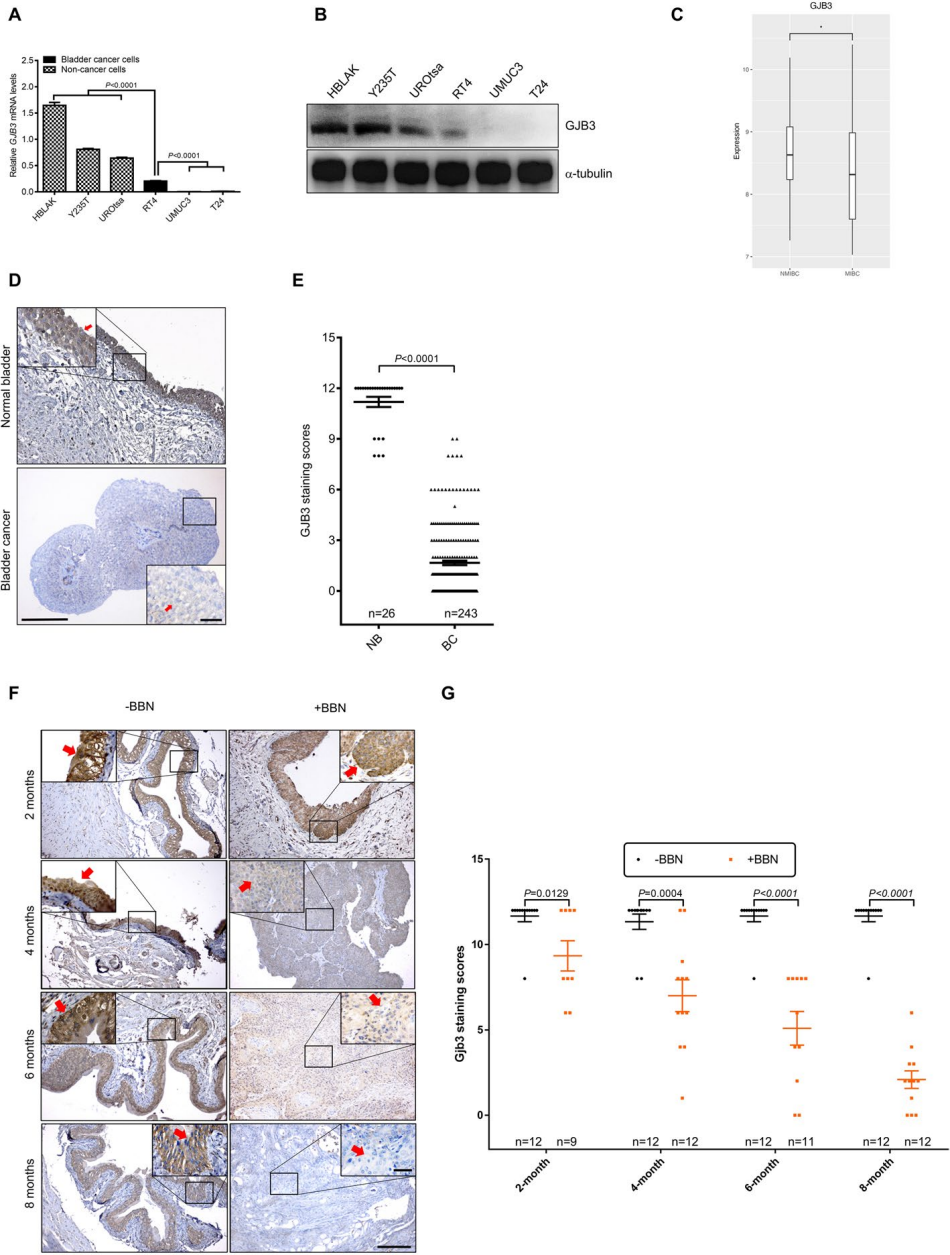
GJB3 as tumor suppressors in bladder cancer. **A–B** (**A**) RT-qPCR was used to measure the GJB3 *mRNA* expression*,* and (**B**) Western blot was employed to ascertain protein amounts. The relative mRNA expression was quantified with respect to *GAPDH*. The qPCR shows results of n = 4 technical repeats. Error bars represent Mean ± SEM. α-tubulin serves as loading control in Western blot. *n* = 3 independent experiments were performed. **C** Comparison of *GJB3* mRNA levels in the CNUH (GSE13507) cohort of NMIBC (*n* = 103) and MIBC (*n* = 62) human bladder tumors. Statistical differences were defined by two-way Fisher’s ANOVA test * = p ≤ 0.05. **D** The IHC images represent the GJB3 staining (red arrows) in human normal bladder and bladder cancer tissues. **E** Quantitation of GJB3-IHC staining scores in human normal bladder and bladder cancer groups. **F** The IHC images display Gjb3 staining (red arrows) in mouse normal bladder and bladder cancer tissues during BBN-induced BC progression. **G** Quantitation of Gjb3-IHC staining scores in normal bladder and bladder cancer tissues during the BBN-induced BC progression in mice. The expression of Gjb3 gradually diminished after the BBN treatment, in contrast to the control mice (black dots), which were given water (orange dots). The bar graph displays mean ± SEM data, and a two-tailed Student's t-test was used to assess significance. Scale bars: 200 μm (**D** and** F**, main panels) and 50 μm (**D** and** F** insets). Images were captured at total magnification of 100 × (**D** and **F** main panels), and 630 × (**D** and** F** insets)

Considering the significant appearance of GJB3 noted in the urothelium and its impact on cell ploidy, the amounts of GJB3 mRNA and protein were ascertained throughout the progression of BC. We observed that GJB3 levels were substantially lower in bladder cancer cells (RT4, UMUC3 and T24) compared to ureter-derived immortalized epithelial cells (HBLAK, Y235T and UROtsa) (Fig. 3A, B). We also noticed that GJB3 expression was lowest in T24 and UMUC3 bladder cells, which have a highly invasive phenotype whereas the expression was more moderate in RT4 cells, which demonstrate low invasiveness. When considered in aggregate with tissue expression data for GJB3 protein (Fig. 1A, B), we demonstrate that GJB3 expression was significantly reduced on bladder cancer cells.

Next, we investigated the possible correlation between GJB3 and bladder cancer progression by comparing *GJB3* mRNA levels in balanced cohorts of NMIBC (*n* = 103) versus MIBC (*n* = 62) human bladder tumors using cohorts from the CNUH dataset (GSE13507) [45]. GJB3 expression was significantly decreased in the MIBC tumors compared to NMIBC tumors (Fig. 3C). GJB3 protein expression in human and mouse bladder samples was further assessed by immunohistochemistry (IHC) utilizing tissue microarrays (TMA), which included 26 normal bladder tissue samples and 243 human MIBC tumor samples. We found reduced GJB3 protein expression in human MIBC samples compared to normal bladder tissue samples. (Fig. 3D, E; see Fig. S2 for more details on MIBC cohort). Similarly, we found lower Gjb3 protein levels during tumor progression in the N-butyl-N-(4-hydroxybutyl)-nitrosamine (BBN)-induced mouse BC model (Fig. 3F, G). Of note, the tumours in this mouse model represent in majority invasive squamous cell carcinoma and represent a high degree of genome instability [46]. Please note that details on tumor progression in the mouse model of BBN-induced BC can be found in our recent publication [31]. In brief, mice were treated with the carcinogen BBN and their bladders were examined at 2, 4, 6, and 8 months after the BBN treatment histologically. We observed early histological changes at 6–8 weeks of BBN-treatment, and all BBN-treated mice developed invasive BC at 8 months BBN-treatment. The immunohistological evaluation of Gjb3 expression in these mice revealed an about 20% reduction of Gjb3 protein levels as early as two months BBN post-treatment (Gjb3 staining scores: 11.7 in -BBN group vs 9.3 in + BBN group, *P* = 0.0129) (Fig. 3G, 2 month time-points). At the four months BBN-treatment group, the overall expression of Gjb3 protein was reduced by more than 35% compared to the control group (Gjb3 staining scores: 11.3 in -BBN group vs 7.0 in + BBN group, *P* = 0.0004) (Fig. 3G, 4 month time-points). Notably, Gjb3 protein levels were reduced by more than 50% after six months of BBN treatment (Gjb3 staining scores: 11.7 in -BBN group vs 5.1 in + BBN group, P < 0.0001) (Fig. 3G, six month time-points), and by 80% after eight months of BBN treatment (Gjb3 staining scores: 11.7 in -BBN group vs 2.1 in + BBN group, *P* < 0.0001) (Fig. 3G, eight month time-points), respectively.

### GJB3 impairs invasion and migration capacities of BC cells

**Fig. 4:**
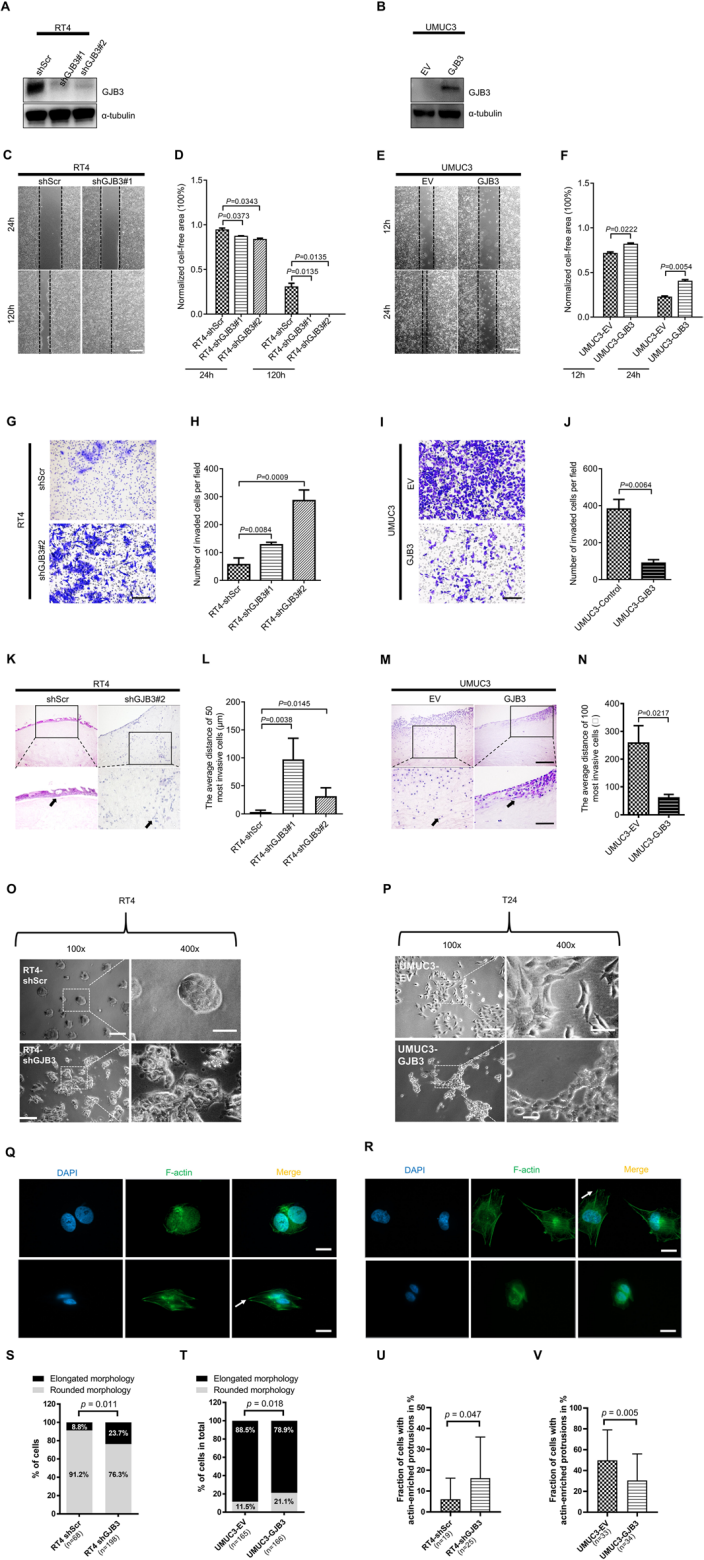
GJB3 inhibits cells invasion and migration.** A**–**B** Western blots displaying the GJB3 protein quantity assessments in RT4 and in UMUC3 cells with experimental modifications. The loading control was provided by α-tubulin levels. The Western blots were repeated for three times.** C** Cell migratory capacity in RT4 cell line with shGJB3#1 was detected by Wound healing/scratch. The exemplary imaged were captured at 24 and 120 h. **D** Normalized cell free area was used to quantify the impact of GJB3 knockdown on RT4 cells by Wound healing assay. *n* = 3 distinct experiments. The bar graphs display mean ± SEM values, and a two-tailed Student's t-test was used to assess significance. **E** Cell migratory capacity in UMUC3 cell line with ectopic GJB3 was detected by Wound healing/scratch. The representative pictures captured at 12 and 24 h in case of UMUC3 cells. **F** Quantitation of normalized cell free area of UMUC3 cells with ectopic GJB3 performed by Wound healing assay* n* = 3 distinct experiments. The bar graphs display mean ± SEM values, and a two-tailed Student’s *t*-test was used to assess significance. **G** The invasion capacity of RT4 cell line with shGJB3#1 was detected by Boyden chamber. The exemplary images depict cell invasion through the Boyden chamber, stained at 144 h post-seeding. **H** Quantitation of invasive capacity of RT4 cells expressing the indicated shRNAs targeting GJB3. *n* = 3 distinct experiments. The bar graphs display mean ± SEM values, and a two-tailed Student’s *t*-test was used to assess significance. **I** The invasion capacity of UMUC3 cell line with ectopic of GJB3 was detected by Boyden chamber. The exemplary images depict cell invasion through the Boyden chamber, stained at 48 h post-seeding. **J,** Quantitation of invasive capacity of UMUC3 cells with ectopic GJB3 expression. *n* = 3 distinct experiments. The bar graphs display mean ± SEM values, and a two-tailed Student's t-test was used to assess significance.** K** Representative images of hematoxylin/eosin stainings showing the invasion capacity of RT4 cell line with shGJB3#2 by porcine bladder ex vivo organ culture method (the invasive capacity of BC cells in the ex vivo organ culture model was quantified as shown in Fig. S4). The cells were seeded on the surface of the de-epithelized porcine bladder for 21 days. **L** Quantitation graphs displaying the impact of GJB3 alteration on the invasive capacity of RT4 cells in ex vivo organ culture approach. *n* = 4 (RT4-shScr), *n* = 3 (RT4-shGJB3#1), *n* = 3 (RT4-shGJB3#2) **M** Representative images of hematoxylin/eosin stainings showing the invasion capacity of UMUC3 cell line with ectopic GJB3 by porcine bladder ex vivo organ culture approach. The cells were seeded on the surface of the de-epithelized porcine bladder for or 14 days. Insets: enlarged images of the areas shown by black boxes. Black arrows indicate the cells that have spread the farthest from the surface. *n* = 3 (UMUC3-EV), *n* = 4 (UMUC3-GJB3) independent experiments were performed. Mean ± SEM values are shown in the bar graph, and significance was determined by two-tailed Student’s *t*-test. Scale bars: 200 μm (**C**, **E**, **G**, **I**, **K** main panels, **M** main panels) and 100 μm (**K** insets, **M** insets). Images were captured at total magnification of 50 × (**C**, **E**), 100 × (**G**, **I**, **K** main panels, **M** main panels), and 200 × (**K** insets, **M** insets). **O**-**V** Morphological changes and actin-enriched protrusions in UMUC3, and RT4 cells with altered GJB3 expression. Cells with actin-enriched protrusions are marked with white arrows. **O**-**P** Brightfield microscopy images depict cells exhibiting a transition towards a round morphology (**O**) of UMUC3 cells with ectopic GJB3 expression. **P** RT4 cells with GJB3 knockdown demonstrate an elongated shape. Magnifications indicate 100 × or 400 × , respectively. The scale bars refer to 100 μm (left), and 50 µm (right). **Q**-**T** Quantification of round or elongated morphology on fixed cells. A cell with elongated or round morphology is identified by the ratio of longest and shortest diameter of the cell from images captured randomly at 630 × magnification using a Zeiss TCS SP5 confocal microscope. Scale bars: 20 μm. The ratio is calculated as the longest diameter of the cell dividing by the shortest diameter of the cell. The ratio is calculated as longest diameter dividing by shortest diameter. A cells with Ratio ≤ 2 is identified as round morphology, while ratio > 2 is elongated morphology. **Q**, **R** Immunofluorescence staining photos illustrate the round morphology shifting of (**Q**) UMUC3 cells with GJB3 overexpression compared to cells transfected with empty vector (EV). **R** RT4 cells with GJB3 knockdown display a transition towards an elongated morphology compared to cells transfected with shScramble (shScr). Cell nuclei are stained with DAPI, and F-actin is labeled with Phalloidin-AF488. **S**, **T** The bar graphs reveals percentages of cells with different morphology in total in each group, shown above in **Q** and **R**. The percentage was calculated as number of cells with elongated (or rounded) morphology divide cell numbers in total. The percentage values in different groups are marked above or in the bars. Black bars indicate percentages of cells with elongated morphology, and gray bars indicate the percentage of cells with rounded morphology. The statistical significance is calculated by using chi-square statistic. **U**, **V** The graphs show the fraction of cells with actin-enriched protrusions in response to GJB3 alterations (**U**) in RT4 or (**V**) in UMUC3 cells. For each picture, the percentage of cells with actin-enriched protrusions is calculated by the number of the cells with actin-enriched protrusions divided by total number of cells. (n) indicates the number of pictures taken in the group

In order to acquire additional insight into the role of GJB3 in bladder cancer progression, GJB3 expression was knocked down in RT4 cells, which inherently have a low invasive potential and migratory capacity. Correspondingly, UMUC3, a cell line with high invasion and migration capacity, was selected for ectopic expression of GJB3. Western blot analysis verified the effective knockdown and ectopic expression of GJB3 in the corresponding cell lines (Fig. 4A, B). Utilizing wound healing assays, we observed that reduced expression of GJB3 significantly enhanced the migration ability of the RT4 cells, whereas ectopic GJB3 in UMUC3 cells had the opposite impact (Fig. 4C–F). Because no changes in cell numbers were detected in response to altered GJB3 levels, we concluded that these factors had a true impact on cell migratory capacity and were not caused by changes in cell proliferation (Fig. S3).

We then used the Boyden chamber method to assess the effect of GJB3 on cell invasion and migration. The exemplary images and evaluation of the results similarly reveal that depletion of GJB3 increases the migratory and invasive competence of the RT4 cells, whereas overexpression of GJB3 reduces these abilities in UMUC3 cells (Fig. 4G–J). Apart from the Boyden chamber experiment conducted in vitro, the influence of GJB3 on the invasive capabilities of RT4 and UMUC3 cells was assessed using the ex vivo porcine organ culture approach (Fig. 4K–N). Representative images show that control cells (RT4-shScr) acquired an epithelium-like structure on the surface of the de-epithelized porcine bladder tissue, whereas RT4-shGBJ3 cells infiltrated the stroma and muscle of the bladder tissue in culture (Fig. 4K). We evaluated the impact of GJB3 on tissue invasion by measuring the distance from the tissue surface to the invasive front (see Fig. S4 for the measurement). The invasive ability of RT4 cells was improved by 8 to 27-fold (3.4 µm with RT4-shScr versus 97.2 µm with RT4-shGJB3#1, *P* = 0.0038, and 31.2 µm with RT4-shGJB3#2, *P* = 0.0145) when was GJB3 knockdown, while GJB3 overexpression impaired the ability of UMUC3 cells to invade into the tissue by 76% as compared to UMUC3-EV (257.6 µm with UMUC3-EV versus 59.9 µm with UMUC3-GJB3, *P* = 0.0217 (Fig. 4L, N).

The above experiments revealed that GJB3 influences migration and invasion capacities of BC cell lines. These features may be associated with changes in cell morphology and epithelial mesenchymal transtion (EMT). Expression of three different genes, (*EpCAM*, the epithelial cell adhesion molecule; *CDH1*, coding for E-cadherin; and *VIM*, coding for vimentin) were assessed by RT-qPCR in order to examine potential EMT transition in response to GJB3 alterations. We did not find significant GJB3-dependent changes in the mRNA levels of these genes (Fig. S5). However, two different experimental settings revealed GJB3-dependent morphology changes in RT4 and UMUC3 cells (Fig. 4O–T). First, phase contrast microscopy in 2D cell culture of living cells was used to inspect the cellular morphology changes associated with GJB3 expression. We observed that knockdown of GJB3 in RT4 cells induced a change from rounded to elongated morphology (Fig. 4O). Conversely, ectopic GJB3 expression in UMUC3 cells resulted in a change of morphology towards a rounded morphology compared to those transfected with an empty vector (Fig. 4P). Subsequently, cell morphology changes were visualized on fixed cells with the assistance of F-actin immunofluorescence. RT4 cells with GJB3 knockdown exhibited rising number of cells with elongated morphology relative to those transfected with shScramble and UMUC3 cells overexpressing GJB3 displayed rather a round morphology. The changes in cell morphology were shown to be significant by measuring cell dimensions using images captured randomly at 630 × magnification using a Zeiss TCS SP5 confocal microscope. The results indicated about twofold changes in the number of cells with altered shape (8.8% in RT4 shScr vs 23.7% in RT4-shGJB3, *p* = 0.008) and (11.5% in UMUC3-EV vs 21.1% in UMUC3-GJB3, *p* = 0.018). The changes in cell morphology are associated with reorganization of cellular F-actin, as we find that GJB3 regulates the formation of actin-enriched protrusions. Quantification of the protrusions by F-actin immunofluorescence staining revealed that less actin-enriched protrusions form in the presence of GJB3 in UMUC3 cells and that reduced levels of GJB3 in RT4 cells are associated with elevated levels of actin-enriched protrusions (Fig. 4Q, R, U and V).

### GJB3 regulates invadopodia formation via actin dynamics

**Fig. 5:**
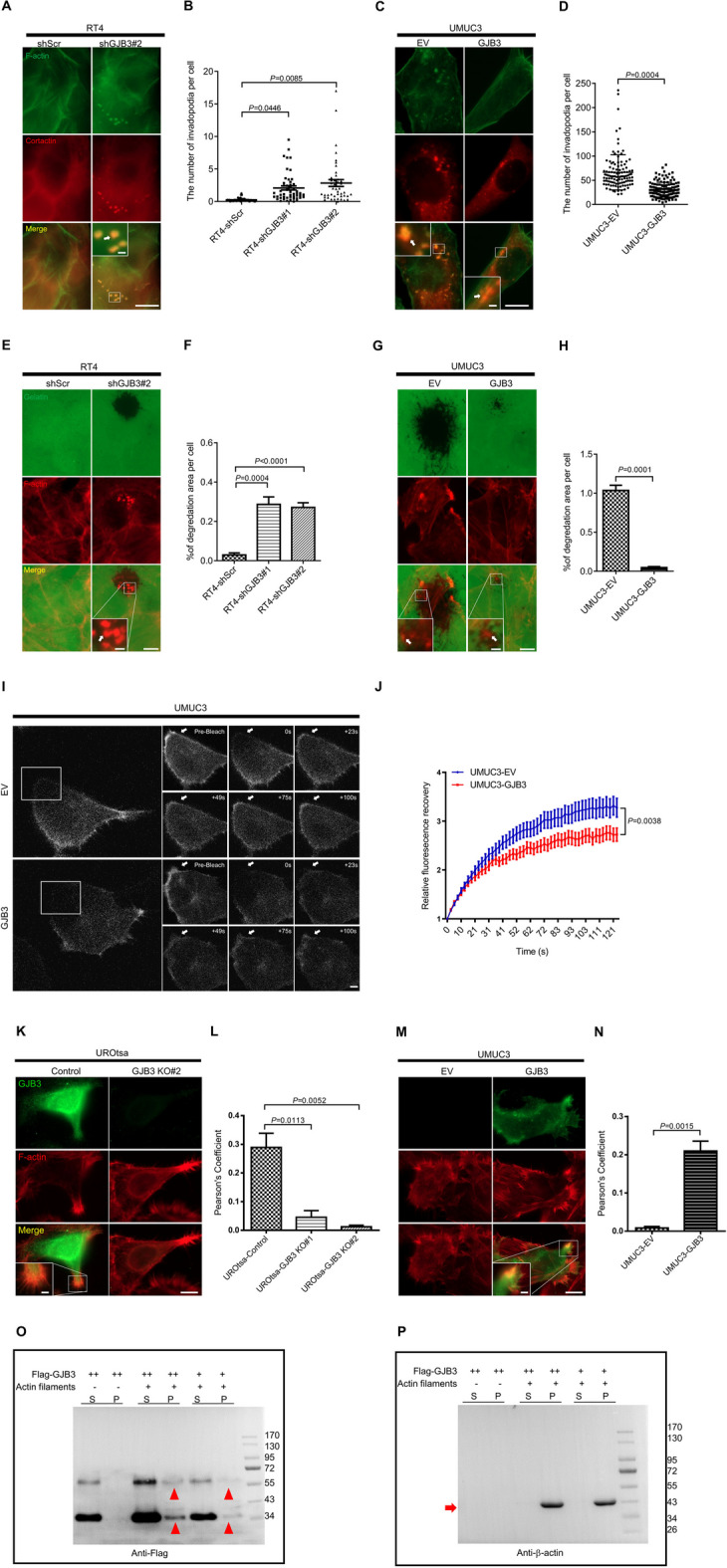
GJB3 interactics with F-actin and influences invadopodia formation via actin dynamics. **A** Representative images demonstrating invadopodia formation of RT4 cells with shGJB3#2. **B** Quantitation of the invadopodia number was performed in RT4 cells. *n* = 959 (RT4-shScr), *n* = 718 (RT4-shGJB3#1), *n* = 481 (RT4-shGJB3#2). Cells are pooled from three independent sets of experiments. **C,** Representative pictures showing invadopodia formation of UMUC3 cells upon ectopic GJB3 expression. **D** Quantitation of the invadopodia number was performed in UMUC3 cells. *n* = 110 (UMUC3-EV), *n* = 137 (UMUC3-GJB3). Cells are pooled from three independent sets of experiments. F-actin is visualized by Alexa Fluor 488-phalloidin and Cortactin is visualized by Cy5, respectively. Yellow spots displaying cortactin and F-actin colocalization identify the invadopodia structures. The regions indicated by white boxes are magnified in the insets. The invadopodia are marked with white arrows. The dot plot displays the mean ± SEM data, and the two-tailed Student's t-test was used to assess significance. **E** Representative pictures indicate the gelatin degradation by RT4 cells upon GJB3 knockdown. **F** Gelatin degradation capacity of the cells was quantified by measuring the degradation area per RT4 cell. *n* = 2809 (RT4-shScr), *n* = 2447 (RT4-shGJB3#1), *n* = 3544 (RT4-shGJB3#2). **G** Representative pictures indicate the gelatin degradation by UMUC3-GJB3 cells. **H** Gelatin degradation capacity of the cells was quantified by measuring the degradation area per UMUC3 cell. *n* = 1048 (UMUC3-EV), and *n* = 1246 (UMUC3-GJB3) are pooled from three to four independent experiments. The dot plot displays the mean ± SEM data, and the two-tailed Student's t-test was used to assess significance. **I** The LifeAct–Ruby signal recovery duration in UMUC3 cells with or without GJB3 is depicted in representative images after LifeAct–Ruby signal bleaching. The white arrows indicate the areas of bleaching. **J**, The quantitation of bleaching recovery experiments with UMUC3 cells. *n* = 29 (UMUC3-EV), *n* = 31 (UMUC3-GJB3). Three different groups of separate experiments' cells are combined. The graphs' data points correspond to the mean ± SEM. P values were calculated using the two-tailed Student's *t*-test at *t* = 49 s.** K** Exemplary pictures displaying the colocalization of GJB3 with F-actin in control UROtsa cells. Insets: enlarged image of the areas shown by white box. **L**, Quantitation of GJB3 and F-actin colocalization in UROtsa by Pearson’s correlation coefficient. *n* = 161 (UROtsa-gControl), *n* = 206 (UROtsa-gGJB3#1), *n* = 276 (UROtsa-gGJB3#2). **M** Exemplary pictures displaying the GJB3/F-actin colocalization in UMUC3-GJB3 cells. Insets: enlarged image of the areas shown by white box. **N** Quantitation of GJB3 and F-actin colocalization in UMUC3 cells by Pearson’s correlation coefficient. *n* = 672 (UMUC3-EV), and *n* = 831 (UMUC3-GJB3) are combined from 3 separate experiments. The two-tailed Student's t-test was used to establish significance, and the bar graph displays mean ± SEM results. Alexa Fluor 647 illustrates the F-actin, and Alexa Fluor 488 illustrates GJB3. Yellow highlights denote GJB3 and F-actin overlap. Insets: enlarged images of the colocalized areas shown by white boxes. **O**, **P** GJB3 binds bundle actin filaments in a dose-dependent manner by Western blot. Actin (2.5 mg/ml) concentrations of GJB3 (relative GJB3 amount is indicated by + or + +). Supernatant (S) and pellet (P) were subjected to 10% SDS-PAGE after high-speed centrifugation at 100,000* g*. Red arrowheads indicate the GJB3, and the red arrow indicates actin filaments visualized by western blot with specific antibodies. *n* = 3 independent experiments were performed. Scale bars: 10 μm (**A**, **C**, **E**, **G**, **K** and **M** main panels), 1 μm (**A**, **C** insets), 2 μm (**E**, **G**, **K** and **M** insets) and 1 μm (**I**). Images were captured at total magnification of 630 × (**A**, **C**, **I**, **K**, **M**) and 400 × (**E**, **G**)

Considering our observation that GJB3 expression intimately regulates cancer cell invasiveness and migratory capacity, we sought to explore the mechanism underlying this relationship, specifically by investigating the impact GJB3 expression has on the function of invadopodia, given their role in cell attachment and remodeling of the extracellular matrix (ECM) during these processes. Using cortactin and F-actin colocalization staining method, we found that RT4 cells with GJB3 knockdown had 7–tenfold more invadopodia formation than RT4-shScr cells (mean values: 0.249 in RT4-shScr versus 2.079 in RT4-shGJB3#1, *P* = 0.0446, and 2.836 in RT4-shGJB3#2, *P* = 0.0085) (Fig. 5A, B). Conversely, ectopic GJB3 expression in UMUC3 cells reduced invadopodia formation ability by 53% compared to that of UMUC3-EV cells (mean values: 66.336 in UMUC3-EV versus 30.620 in UMUC3-GJB3, *P* = 0.0004) (Fig. 5C, D).

Furthermore, we investigated the influence of GJB3 on invadopodia formation at the subcellular level. GJB3 knockdown accelerated gelatin degradation by RT4 cells more than eightfold, seen as black regions on the outside margins and inside of cell islands (mean values: 0.029 with RT4-shScr versus 0.286 with RT4-shGJB3#1, *P* = 0.0004, and 0.271 with RT4-shGJB3#2, *P* < 0.0001) (Fig. 5E, F). In contrast, ectopic GJB3 caused a 95% reduction in gelatin degradation activity of UMUC3 cells compared to UMUC3 cells with an empty vector (mean values: 1.036 with UMUC3-EV versus 0.047 with UMUC3-GJB3, *P* = 0.0001). (Fig. 5G, H).

The regulation of actin dynamics is critical for cell motility, invasion, and invadopodia formation. Thus, we used fluorescence recovery after photobleaching (FRAP) to investigate the effect of GJB3 on peripheral actin dynamics. Quantifying the fluorescence recovery of the F-actin-binding factor LifeAct-Ruby provides a measurement to visualize the actin polymerization [41]. Relative fluorescence recovery following photobleaching of the LifeAct-Ruby signal found in lamellipodia was decreased in UMUC3-GJB3 cells compared to UMUC3-EV cells (Fig. 5I, J). Quantitation of these results revealed that the relative fluorescence signal recovery in the bleaching area was significantly reduced in UMUC3-GJB3 cells compared to UMUC3-EV control cells (2.832 at 62 s, and 3.275 at 124 s with EV versus 2.449 at 62 s, and 2.725 at 124 s with GJB3 overexpression, *P* = 0.0038), suggesting that GJB3 is involved in the assembly process of actin helices.

### GJB3 interacts with F-actin

Considering our above described experimental evidence showing a contribution of GJB3 in invadopodia formation and actin dynamics, we investigated a possible colocalization of GJB3 with F-actin, the main component for cell protrusions. In order to examine potential GJB3 and F-actin colocalization, we used the UROtsa-gGJB3 knockout cells, as introduced and described in Fig. 2, as well as UMUC3 cells with ectopic overexpression of GJB3. As indicated by the co-immunofluorescence staining results, GJB3 signals overlapped with F-actin at the lamellipodia regions of UROtsa cells as well as of UMUC3 cells with ectopic GJB3. In contrast, there was no overlap in UROtsa-gGJB3 cells or UMUC3-EV control cells (Fig. 5K–M). Quantitation of these results by way of Pearson’s correlation coefficient showed that the absence of GJB3 in UROtsa cells caused a 84 to 96% reduction in co-localization with F-actin with two different gRNAs compared to control cells (*r* = 0.289 in UROtsa-control versus 0.045 in UROtsa-gGJB3#1, *P* = 0.0113, and *r* = 0.012 in UROtsa-gGJB3#2, *P* = 0.0052) (Fig. 5L). Alternatively, overexpression of GJB3 in UMUC3 increased colocalization with F-actin by 25-fold when compared to control cells with an empty vector (*r* = 0.008 with UMUC3-EV versus r = 0.210 with UMUC3-GJB3, *P* = 0.0015) (Fig. 5N). We further explored the suspected interaction of GJB3 with F-actin by co-sedimentation assays and could demonstrate that GJB3 protein can directly bind to polymerized actin in a dose-dependent manner, as indicated by the Western blot approach (Fig. 5O, P).

In order to investigate the relationship between GJB3 and F-actin during cell migration, we next conducted GJB3/F-actin co-immunostaining using T24 BC cell line in the scratch assay. While F-actin is distributed in the cell matrix in during the migration of control cells (T24-EV) with protruding cell extensions in the direction of cell migration (in the front line of cells), GJB3 expressing T24 cells exhibit a rather flat surface and accumulate F-actin at the migration side, showing some GJB3/F-actin colocalization (Fig. S6A). This is intruiging as Bisaria et al. (2020) recently reported that during migration, membrane-proximal F-actin (MPA) locates at the opposite site of cell migration direction [47]. They used a synthetic F-actin-CaaX fusion protein (MPact), which selectively localizes to the plasma membrane and quickly diffuses at ~ 1 μm2/s and allows to distinguish and track dynamic changes of F-actin density. We received the MPact vector from the authors and investigated GJB3's role during cell migration on F-actin polymerization using MPAct dynamics. GJB3 overexpression caused MPAct enrichment at the front leading edge and impaired the formation of cell protrusions at the migration surface (Fig. S6B), indicating that GJB3 may impair the cell migration by enhanced MPA formation. In summary, these results indicate that GJB3 influences F-actin dynamics and/or reorganization in response during cell migration.

## Discussion

Using genome-wide RNAi screening methodology, our previous work has identified GJB3 as a sentinel gene that regulates cell ploidy [1, 2]. Remarkably, mouse xenograft studies revealed that, GJB3 deficiency promotes tumorigenic transformation of RB and p53 check-point deficient, telomerase-immortalized BJ fibroblasts into tumor cells [2]. The aim of the current study was to explore the molecular mechanism by which the loss of GJB3 leads to aneuploidy-induction in urothelial cells and the role of GJB3 downregulation in the development and progression of urothelial carcinoma of the bladder. Here, we demonstrate that ureter and bladder epithelial cells highly express the GJB3 protein and further, that the expression of this protein plays an important role in maintaining chromosomal stability. This results support our previous findings that GJB3 loss may play a role in the development of malignant transformation during tumorigenesis. In line with this idea, we find that expression of GJB3 is downregulated in human and mouse bladder cancer cells lines as well as in bladder cancer tissue samples derived from patients at time of the trans urethral resection of bladder tumor. Bladder tumors analyzed from BBN-induced spontaneous bladder cancer murine models similarly confirmed reduced levels of GJB3 in comparison to normal bladder tissue, implicating a role of reduced GJB3 expression in bladder carcinogenesis. Our study additionally associates the reduced GJB3 expression with increased invasive and migratory characteristics of cells. Indeed, we found that downregulation of the GJB3 expression enhanced the dynamic polymerization of actin to form actin-rich cell protrusions, called invadopodia, and thus conferred a more invasive phenotype to urothelial cancer cells, contributing to the progression of bladder cancer to the MIBC phenotype.

Mechanistically, the results of our immunofluorescence and binding experiments reveal discernable interactions between GJB3 and cytoskeleton proteins α-tubulin and F-Actin, and further, the moderation of microtubule assembly and actin polymerization with preserved or overexpression of GJB3. Deficiencies in GJB3 expression were noted to exaggerate microtubule and actin assembly, events that were shown to cause instability in the microtubule spindle apparatus during mitosis and increase the generation of invadopodia, respectively. Consequently, GJB3 deficiency leads to spindle orientation defects, cytokinesis failure and centrosome amplification resulting in aneuploidy. Notably, our research further sheds light on the integral role of GJB3 in the mediating crosstalk between microtubules, important components of the mitotic spindle apparatus, and the actin cytoskeleton, which is involved in cellular motility and migration. When considered in aggregate, these data support our hypothesis that altered GJB3 protein function will compromise the genomic stability of urothelial cells and enhance the invasive potential of bladder cancer cells contributing to metastatic progression.

The significance of this observation in the context of cancer research lies in the molecular link we identify between development of cellular aneuploidy and the cancer cell metastatic cascade. The results of our study suggest that adversely impacting factors associated with the regulation of the cellular cytoskeleton components and their dynamic functions may in turn cause both cell aneuploidy and cell invasiveness and migration during tumor progression. Conventionally, metastatic progression of cancer cells, often a late event in carcinogenesis, has been causally linked to the development of cellular aneuploidy during earlier stages of malignant transformation. It has been postulated that aneuploidy results in continuous alterations in the genomic background of cells, with in turn can eventually cause instability in genes related to cell migration and confer an invasiveness phenotype [48]. However, studies exploring this hypothesis of a stepwise progression from aneuploidy to cellular invasion remain contradictory and fail to corroborate the relationship between aneuploidy and metastasis. For example, Anand et al. find the gaining chromosome 5 can increase invasion, migration, and motility in HCT116 cells, while in RPE1 cells, single chromosome 5 gaining will suppress metastatic behavior [49]. Therefore, it may follow based on our study findings, that heterogeneity exists in the process of malignant transformation in some cancer states. Specifically, we highlight that the dysregulation of ploidy-control genes can promote invasion and migration in a simultaneous fashion, rather than through a stepwise progression wherein aneuploidy precedes cell invasion. In this sense, the increased aneuploidy found in metastatic cells may rather be a consequence of mutations in genes that promote invasiveness during tumor progression.

Of note, we have recently reported that the knockdown or loss of Oxysterol-binding protein-related protein 3 (ORP3), which was identified as another ploidy-control gene in the above-mentioned RNAi screen, also interacts with cytoskeletal components, F-actin and γ-tubulin, respectively. ORP3 downregulation also influences actin as well as microtubule dynamics, induces aneuploidy in Y235T cells, enhances invadopodia formation and promotes the invasive ability of bladder cancer cells. Moreover, we observed decreased ORP3 levels in human BC and BBN-induced mouse BC, and Orp3-KO mice are more prone to developing BBN-induced BC [31]. In the same line, we have recently described the role of TKS5 (SH3PXD2A) in simultaneously regulating cell mitosis in addition to cellular invadopodia formation. Specifically, deficiency of TKS5, an important factor in promoting actin filament polymerization, was observed to not only confer a more invasive phenotype for cells, but also induce mitotic defects [32]. As such, the association often observed between elevated cellular aneuploidy and higher metastatic potential of cancer cells may reflect the existence of underlying cellular processes that undermine the cytoskeletal functionality of the cells and which not only interfere genomic integrity, but also directly impact their invasiveness potential through invadopodia formation.

There is sound scientific rationale to support our demonstration of the dual role of actin dynamics in processes affecting cell motility and, cell division, particularly given that the interaction between actin cytoskeleton and microtubules is widely recognized in the cell biology literature. Indeed, proper spindle position and orientation, essential for both cell mitosis and migration, requires intimate crosstalk between the actin cytoskeleton and microtubules [50]. Actin is an essential constituent of the cytoskeleton and is involved in many aspects of cellular dynamics, including invasion and motility of cells. Cellular processes such as cell migration and division depend on actin polymerization, which is the tightly controlled assembly of actin monomers into filamentous structures. It is intruiging that polymerization of membrane proximal F-actin (MPA) accumulates at the surface of GJB3-expressing T24 cells in the wound healing assay (Fig. S6). As indicated above, Bisaria et al. (2020) recently found that F-actin is enhanced at the cell front in cells with increased migratory capacity while MPA is increased in the rear of the cell [47]. Interestingly, we observe the colocalization of GJB3 and F-actin in the actin enrichment area. We conclude that MPA enrichment by GJB3 establishes a border in front of the cells which can block cell migration. This result is in line with previous reports. Welf et al. (2020) have shown that the cell protrusion needs the depletion of actin membrane, not only upregulation of actin polymerization [51]. We think that GJB3 helps to stabilize MPA and decreases the actin dynamic turnover. This idea is supported by findings reported by Ilić et al. (1995), showing that actin stabilization can inhibit the rapid turnover, which can lead to decreased cell motility despite increased actin filament density at specific locations [52]. In the same line, interaction of actin-regulating proteins and actin filaments can lead to the stabilization of actin at the cell front despite overall reduced polymerization dynamics [53]. Considering that GJB3 expression attenuates invadopodia formation (Fig. 5), actin-enriched protrusions at the leading edge of cells, we speculate that GJB3 impacts cell migration and mitosis by influencing actin filament dynamics and reorganization.

During cell mitosis, the actin cytoskeleton adopts a structure of high cortical rigidity via dynamic polymerization so as to provide an anchor and pulling force for astral microtubules, which can then orient the mitotic spindles [54]. Furthermore, actin polymerization at the leading edge of migrating cells generates filopodia and lamellipodia, two types of membrane protrusions that aid in cell migration across the extracellular matrix [55]. Similarly, microtubule dynamics is integrally associated with cell motility and invasion, having a major impact on cellular behavior of in health and disease. During cell migration, microtubules realign and move toward the leading edge, facilitating targeted motility. Furthermore, microtubule dynamics influences the generation and turnover of focal adhesions, which are critical structures that tie the cell to the extracellular matrix during migration [56]. Thus, deregulated microtubule dynamics influences increased invasiveness and metastatic potential of cancer cells by regulating cytoskeletal reorganization, cell adhesion, and proteolytic enzymatic activity. Microtubules have also been shown to reciprocally manipulate cellular processes regulated by the actin cytoskeleton [57]. Specific examples of this dynamic include the recognized need for persistent microtubule assembly to elongate and hold long actin-rich cell protrusions during invasion [58], and role the microtubule organizing center (e.g. centrosome) plays in polarizing cells and maintain their directionality during cellular movement [48]. While the molecular mechanisms underlying the crosstalk between the actin cytoskeleton and microtubules remain yet to be fully characterized, the results from our current study suggest that the GJB3 protein may have an intermediary role herein through direct interactions with microtubules spindles and actin filaments, respectively.

The clinical relevance of this hypothesis, which is supported by our work, pertains to the conceivable role GJB3 dysregulation has on inducing cellular aneuploidy and enhancing migratory/invasive capacity of bladder cancer cells—features intimately associated with the malignant transformation of cells and their enhanced metastatic potential. In fact, GJB3 expression is significantly reduced during bladder cancer progression in both humans and murine models, with near complete loss of GJB3 expression in highly invasive stages of disease. This was confirmed through our in vitro experiments, which show that the loss of GJB3 contribute not only to the development of cellular aneuploidy, but also promotes development of a highly invasive phenotype. Interestingly, a similar association between another connexin protein and cellular genomic stability and migratory capacity has been noted in hepatocellular carcinoma cells. Specifically, studies in hepatocellular carcinoma cells reveal that the dysregulation of GJA1 (Cx43) expression can enhance cell invasion and migration [59] as well as disrupt normal cell mitosis [60]. Moreover, using co-immunoflourescence and co-immunopreciptation experiments, multiple interactions between GJA1 and cytoskeleton components, e.g. drebrin or tubulin, was demonstrated [61]. The striking similarities noted regarding the impact of different connexin proteins, namely GJB3 and GJA1, on cellular processes across different cancer cell types further supports our hypothesis that dysfunction of cytoskeleton-associated genes can influence in parallel both cell ploidy and migratory/invasive capacity.

## Conclusions

The findings presented in this study demonstrate a molecular link between elevated aneuploidy and tumor cell invasion, two crucial features of metastatic cells. Along with our recent reports on ploidy-control and cell invasion/migration [31, 32], the results presented in this study suggest that altered expression or mutation of many factors that interact with actin and/or microtubule components, affecting tumor cell migration and invasion capacity via the dynamics of these cytoskeleton components, may cause aneuploidy.

Furthermore, the findings convey new insights to our understanding of cancer initiation and progression. As the data point out that the dysfunction of cytoskeleton associated factors may impact cell ploidy, the results also provide a plausible interpretation for the unexpectedly large number of genes and pathways that were identified by several laboratories, which demonstrated that a growing list of chemicals, genetic factors (including oncogenes), and cellular stress factors may cause aneuploidy in eukaryotic cells [62, 63]. In fact, Conery and Harlow [64] argued that the number of aneuploidy-inducing gene alterations could be significantly higher than previously anticipated, including a range of cellular signaling pathways. This idea coincides with our earlier findings from a genome-wide iRNA screening, in which we found a significant number of cancer-associated genes with a novel connection to ploidy control [2]. Secondly, our findings shed light on the debate over whether aneuploidy is a cause or consequence of cancer. While it is clearly documented that aneuploidy and CIN, induced by mutations in genes that control genome integrity, can instigate tumorigenesis, our results support the idea that in later stages of tumor progression, increased aneuploidy may be a consequence of tumor progression, when mutations or altered gene expression provide cancer cells with migratory and invasion capabilities through the dysfunction of the cytoskeletal proteins.

### Supplementary Information

Additional file 1: Supplementary figures and tables: Figure S1. Detection of GJB3 by IHC in human and mouse normal bladder tissues. Figure S2. Clinical data of patients with BC and the evaluation of patient survival. Figure S3. GJB3 has no impact on cell viability and proliferation. Figure S4. Example figure showing the measurement of the invasive capacity of cells in the ex vivo porcine bladder organ model. Figure S5. RT-qPCR for the detection mRNA levels of markers for epithelial-to-mesenchymal transition. Figure S6. Immunoflourescence on fixed cells and MPact live cell imaging for detecting potential colocalization of F-actin and GJB3 during cell migration. Table S1. Cloning primers. Table S2. ShRNA vectors and sequences. Table S3. Guide RNA vectors and sequences. Table S4. Primers for qPCR.

Additional file 2: Supplementary video files on microtubule dynamics in Y235T and UMUC3 cells in response to GJB3 alterations.

## Data Availability

All data analysed during this study to evaluate the conclusions are included within the article or available in supplemental information. Data supporting the results of this study are available from the corresponding author upon reasonable request.

## Abbreviations

BC: Bladder cancer
BSA: Bovine serum albumin
CDKs: Cyclin-dependent kinases
CIN: Chromosome instability
DAPI: 4',6-Diamidino-2-phenylindole
EV: Empty vector
F-actin: Filamentous actin
FITC: Fluorescein isothiocyanate
FRAP: Fluorescence recovery after photobleaching
GJA1: Gap junction alpha 1
GJB2: Gap junction beta 2
GJB3: Gap junction beta 3
GJB5: Gap junction beta 5
IF: Immunofluorescence
IHC: Immunohistochemistry
MPAct: Membrane-proximal F-actin
mRNA: Messenger ribonucleic acid
NSCLC: Non-small cell lung cancer
MIBC: Muscle-invasive bladder cancer
NMIBC: Non-muscle-invasive bladder cancer
ORF: Open reading frame
PCR: Polymerase chain reaction
Rb: Retinoblastoma
RNAi: Ribo nucleic acid (RNA) interference
SEM: Standard error of the mean
SPDF: Spindle pole displacement factor
ORP3: Oxysterol-binding protein-related protein 3
TBS: Tris-buffered saline
WB: Western blot
