# Supplementary figures and images for: Impairment of α-tubulin and F-actin interactions of GJB3 induces aneuploidy in urothelial cells and promotes bladder cancer cell invasion
Source: Cell Mol Biol Lett. 2024 Jul 2;29:94. doi: 10.1186/s11658-024-00609-2 (PMC11218312; doi:10.1186/s11658-024-00609-2)

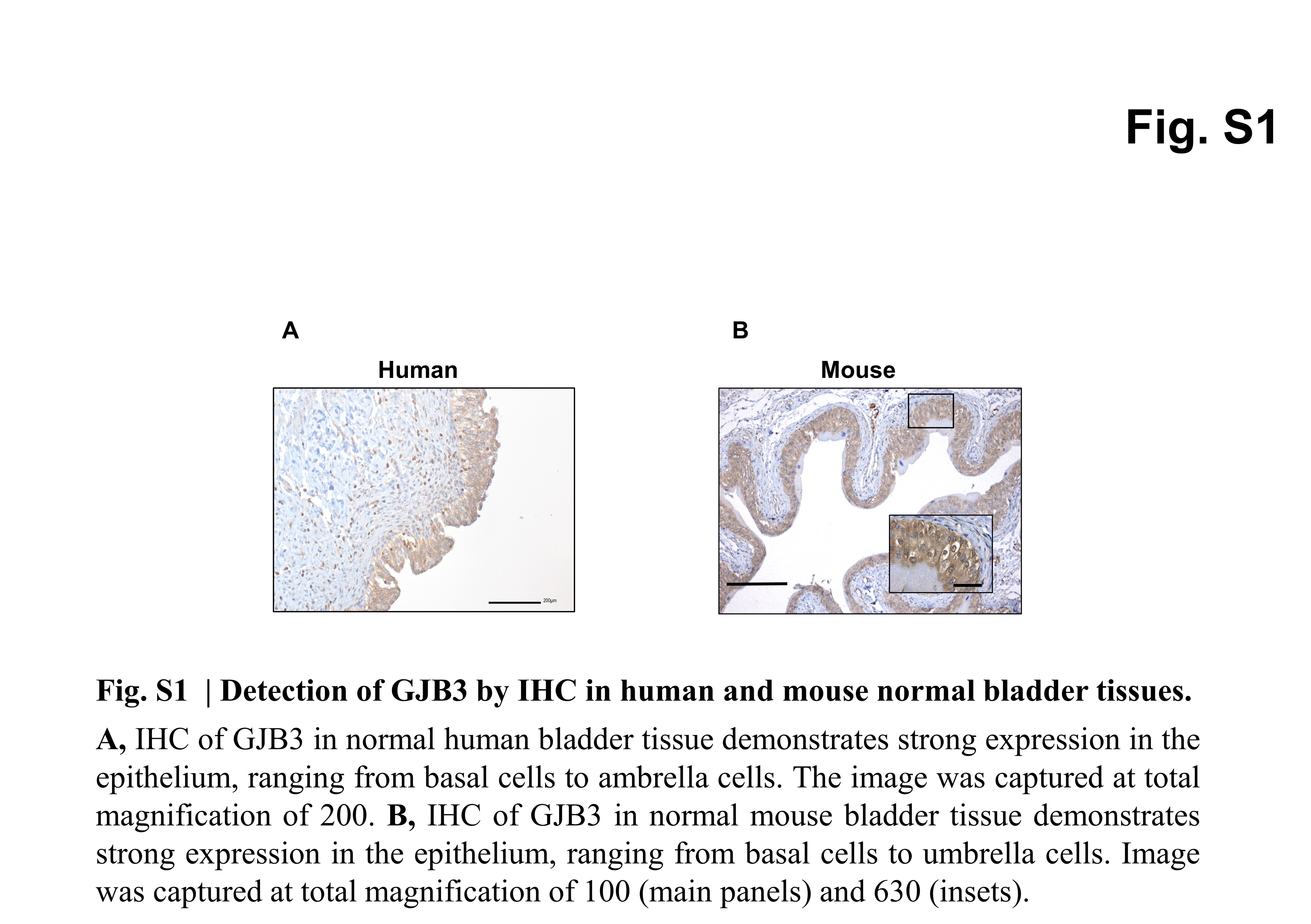

Supplement: Supplementary file 1 — Additional file 1: Supplementary figures and tables: Figure S1. Detection of GJB3 by IHC in human and mouse normal bladder tissues. Figure S2. Clinical data of patients with BC and the evaluation of patient survival. Figure S3. GJB3 has no impact on cell viability and proliferation. Figure S4. Example figure showing the measurement of the invasive capacity of cells in the ex vivo porcine bladder organ model. Figure S5. RT-qPCR for the detection mRNA levels of markers for epithelial-to-mesenchymal transition. Figure S6. Immunoflourescence on fixed cells and MPact live cell imaging for detecting potential colocalization of F-actin and GJB3 during cell migration. Table S1. Cloning primers. Table S2. ShRNA vectors and sequences. Table S3. Guide RNA vectors and sequences. Table S4. Primers for qPCR. [file 11658_2024_609_MOESM1_ESM.zip › Additional file 1/Fig. S1.JPG]

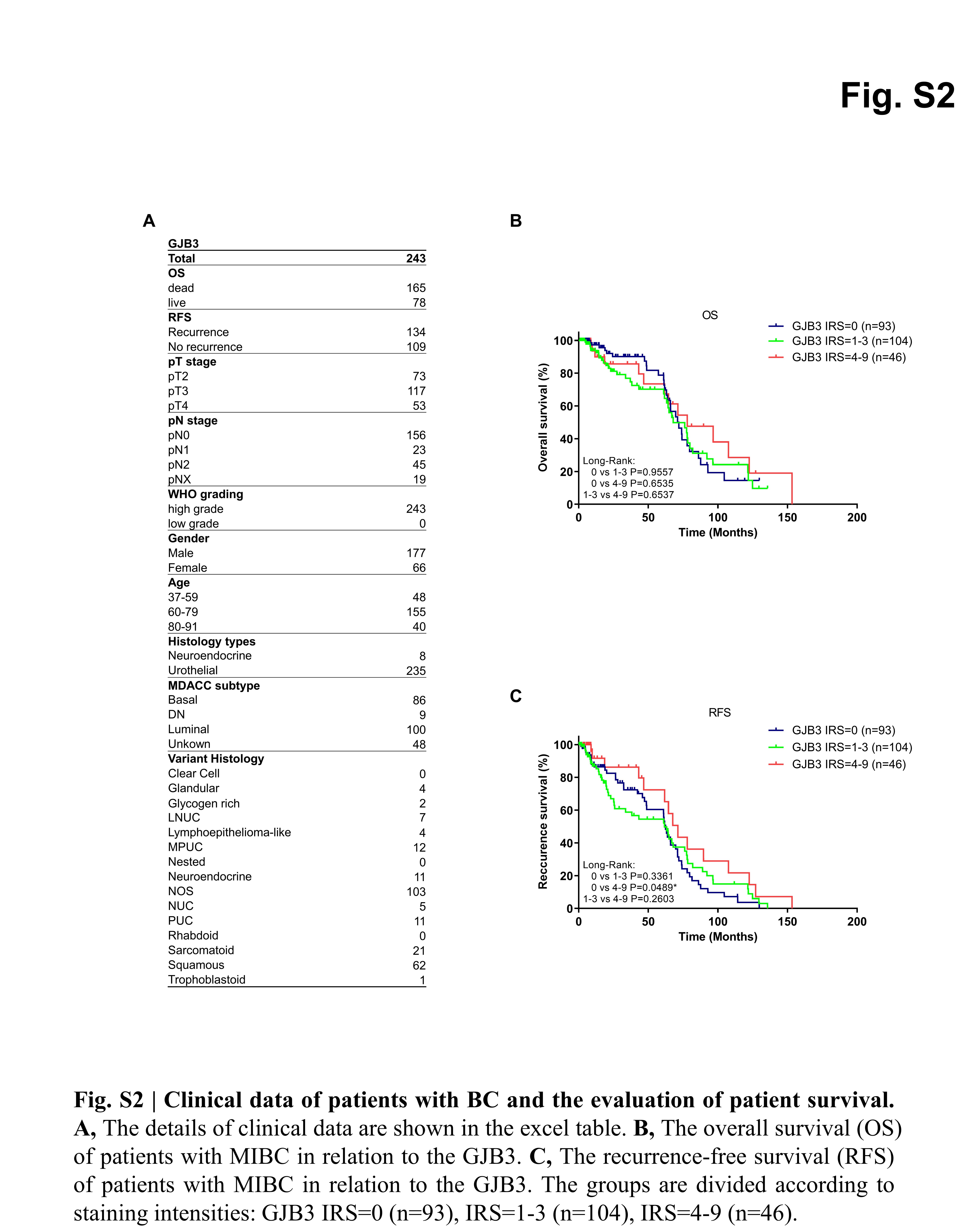

Supplement: Supplementary file 1 — Additional file 1: Supplementary figures and tables: Figure S1. Detection of GJB3 by IHC in human and mouse normal bladder tissues. Figure S2. Clinical data of patients with BC and the evaluation of patient survival. Figure S3. GJB3 has no impact on cell viability and proliferation. Figure S4. Example figure showing the measurement of the invasive capacity of cells in the ex vivo porcine bladder organ model. Figure S5. RT-qPCR for the detection mRNA levels of markers for epithelial-to-mesenchymal transition. Figure S6. Immunoflourescence on fixed cells and MPact live cell imaging for detecting potential colocalization of F-actin and GJB3 during cell migration. Table S1. Cloning primers. Table S2. ShRNA vectors and sequences. Table S3. Guide RNA vectors and sequences. Table S4. Primers for qPCR. [file 11658_2024_609_MOESM1_ESM.zip › Additional file 1/Fig. S2.JPG]

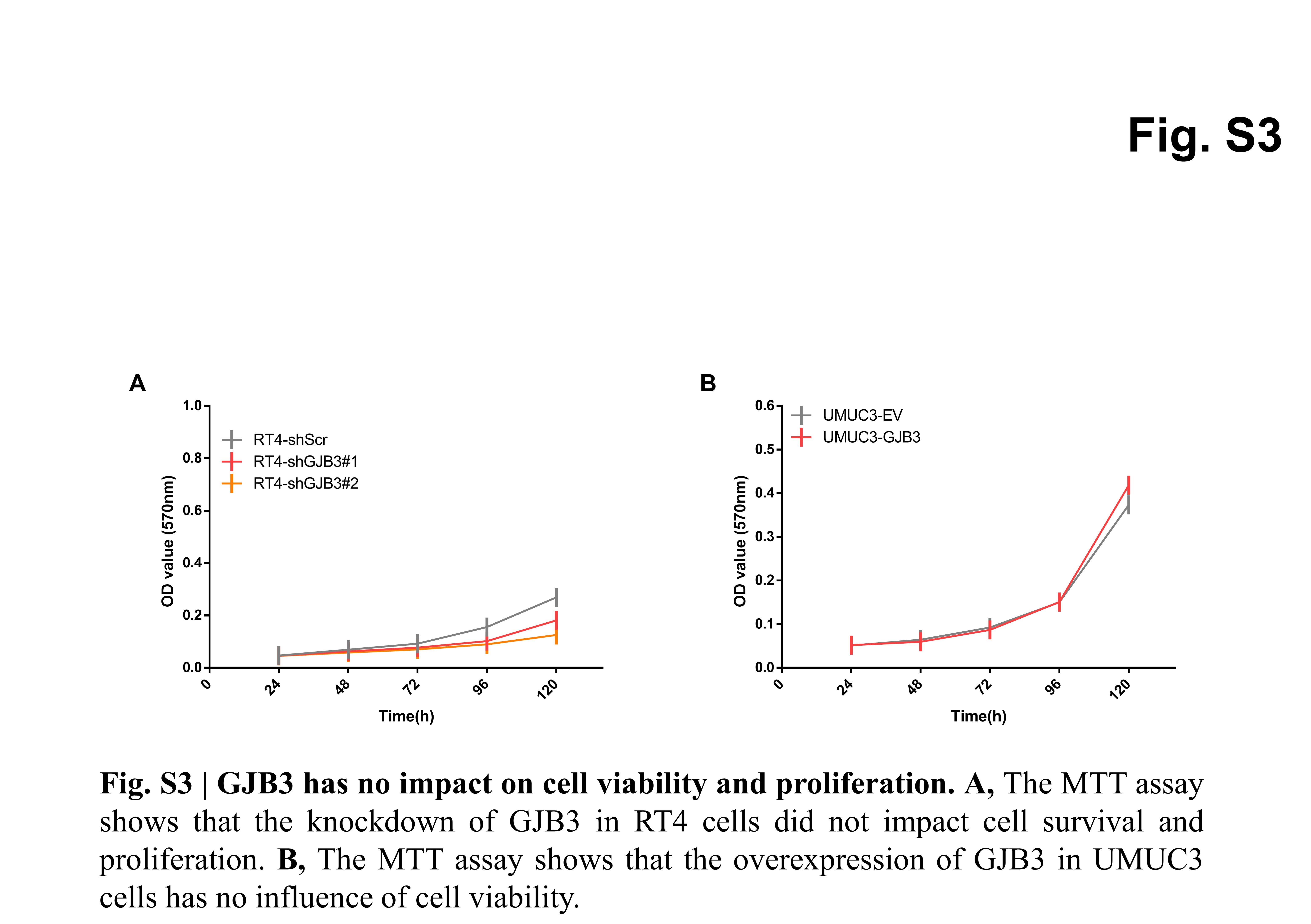

Supplement: Supplementary file 1 — Additional file 1: Supplementary figures and tables: Figure S1. Detection of GJB3 by IHC in human and mouse normal bladder tissues. Figure S2. Clinical data of patients with BC and the evaluation of patient survival. Figure S3. GJB3 has no impact on cell viability and proliferation. Figure S4. Example figure showing the measurement of the invasive capacity of cells in the ex vivo porcine bladder organ model. Figure S5. RT-qPCR for the detection mRNA levels of markers for epithelial-to-mesenchymal transition. Figure S6. Immunoflourescence on fixed cells and MPact live cell imaging for detecting potential colocalization of F-actin and GJB3 during cell migration. Table S1. Cloning primers. Table S2. ShRNA vectors and sequences. Table S3. Guide RNA vectors and sequences. Table S4. Primers for qPCR. [file 11658_2024_609_MOESM1_ESM.zip › Additional file 1/Fig. S3.JPG]

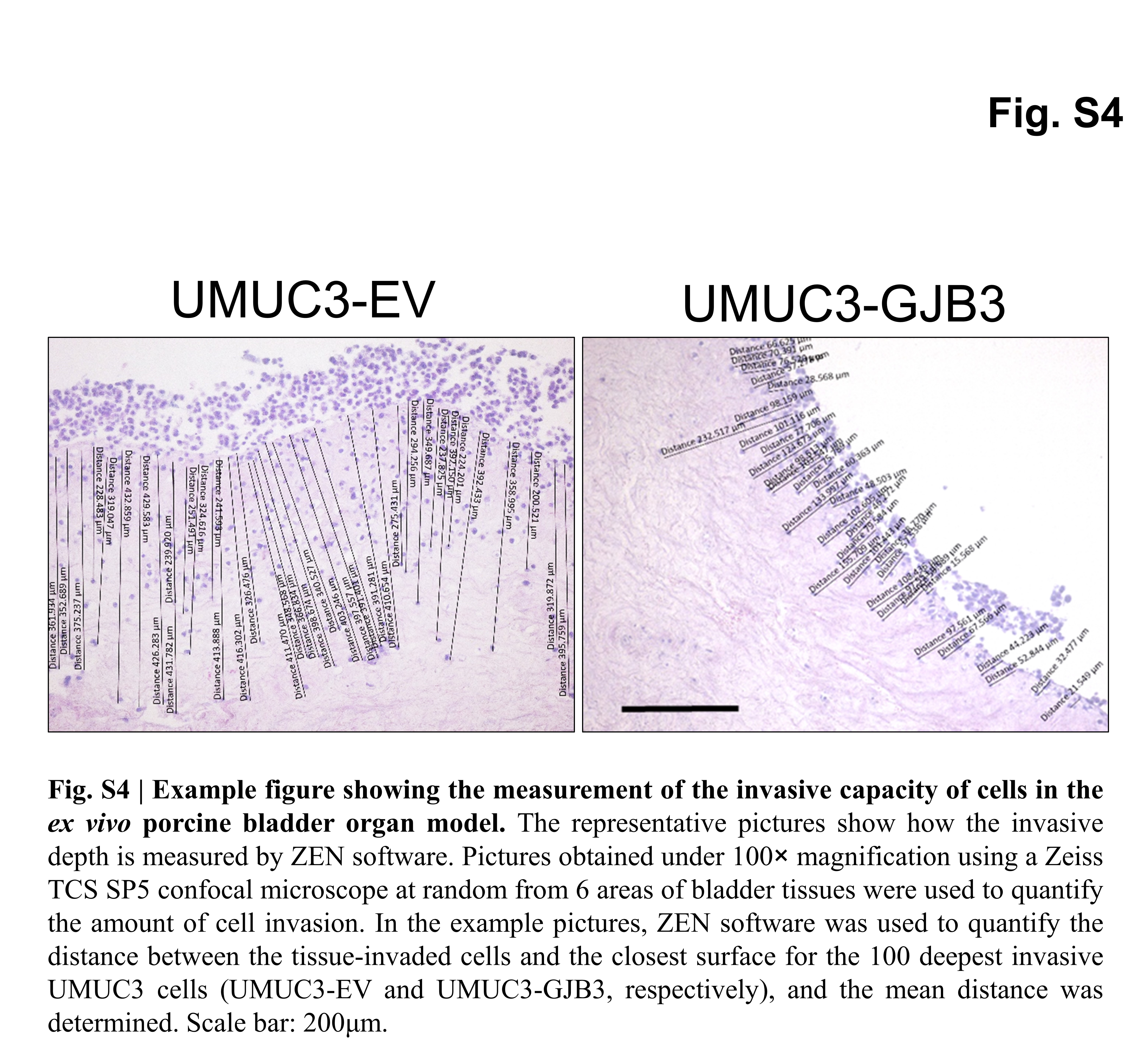

Supplement: Supplementary file 1 — Additional file 1: Supplementary figures and tables: Figure S1. Detection of GJB3 by IHC in human and mouse normal bladder tissues. Figure S2. Clinical data of patients with BC and the evaluation of patient survival. Figure S3. GJB3 has no impact on cell viability and proliferation. Figure S4. Example figure showing the measurement of the invasive capacity of cells in the ex vivo porcine bladder organ model. Figure S5. RT-qPCR for the detection mRNA levels of markers for epithelial-to-mesenchymal transition. Figure S6. Immunoflourescence on fixed cells and MPact live cell imaging for detecting potential colocalization of F-actin and GJB3 during cell migration. Table S1. Cloning primers. Table S2. ShRNA vectors and sequences. Table S3. Guide RNA vectors and sequences. Table S4. Primers for qPCR. [file 11658_2024_609_MOESM1_ESM.zip › Additional file 1/Fig. S4.JPG]

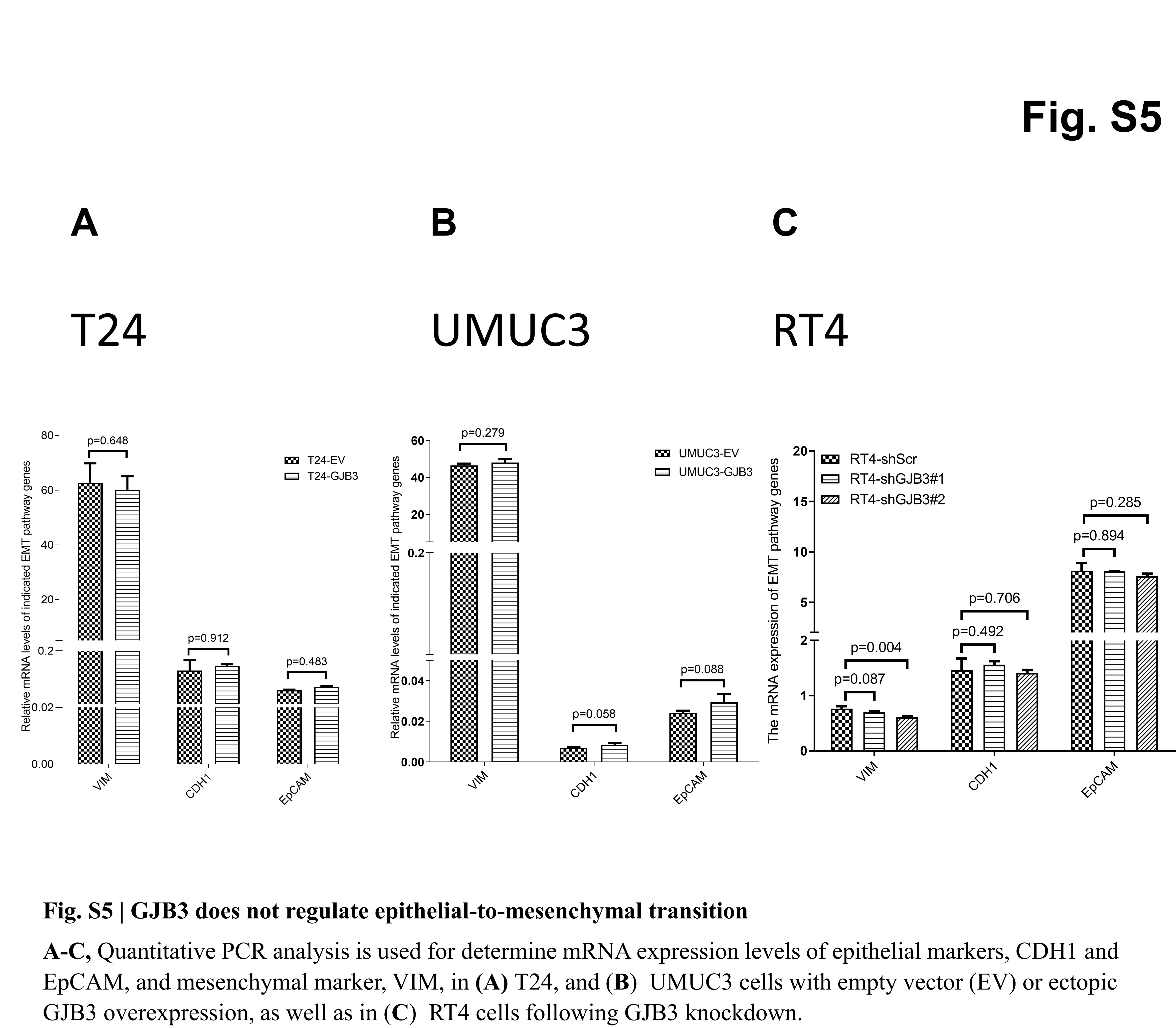

Supplement: Supplementary file 1 — Additional file 1: Supplementary figures and tables: Figure S1. Detection of GJB3 by IHC in human and mouse normal bladder tissues. Figure S2. Clinical data of patients with BC and the evaluation of patient survival. Figure S3. GJB3 has no impact on cell viability and proliferation. Figure S4. Example figure showing the measurement of the invasive capacity of cells in the ex vivo porcine bladder organ model. Figure S5. RT-qPCR for the detection mRNA levels of markers for epithelial-to-mesenchymal transition. Figure S6. Immunoflourescence on fixed cells and MPact live cell imaging for detecting potential colocalization of F-actin and GJB3 during cell migration. Table S1. Cloning primers. Table S2. ShRNA vectors and sequences. Table S3. Guide RNA vectors and sequences. Table S4. Primers for qPCR. [file 11658_2024_609_MOESM1_ESM.zip › Additional file 1/Fig. S5.JPG]

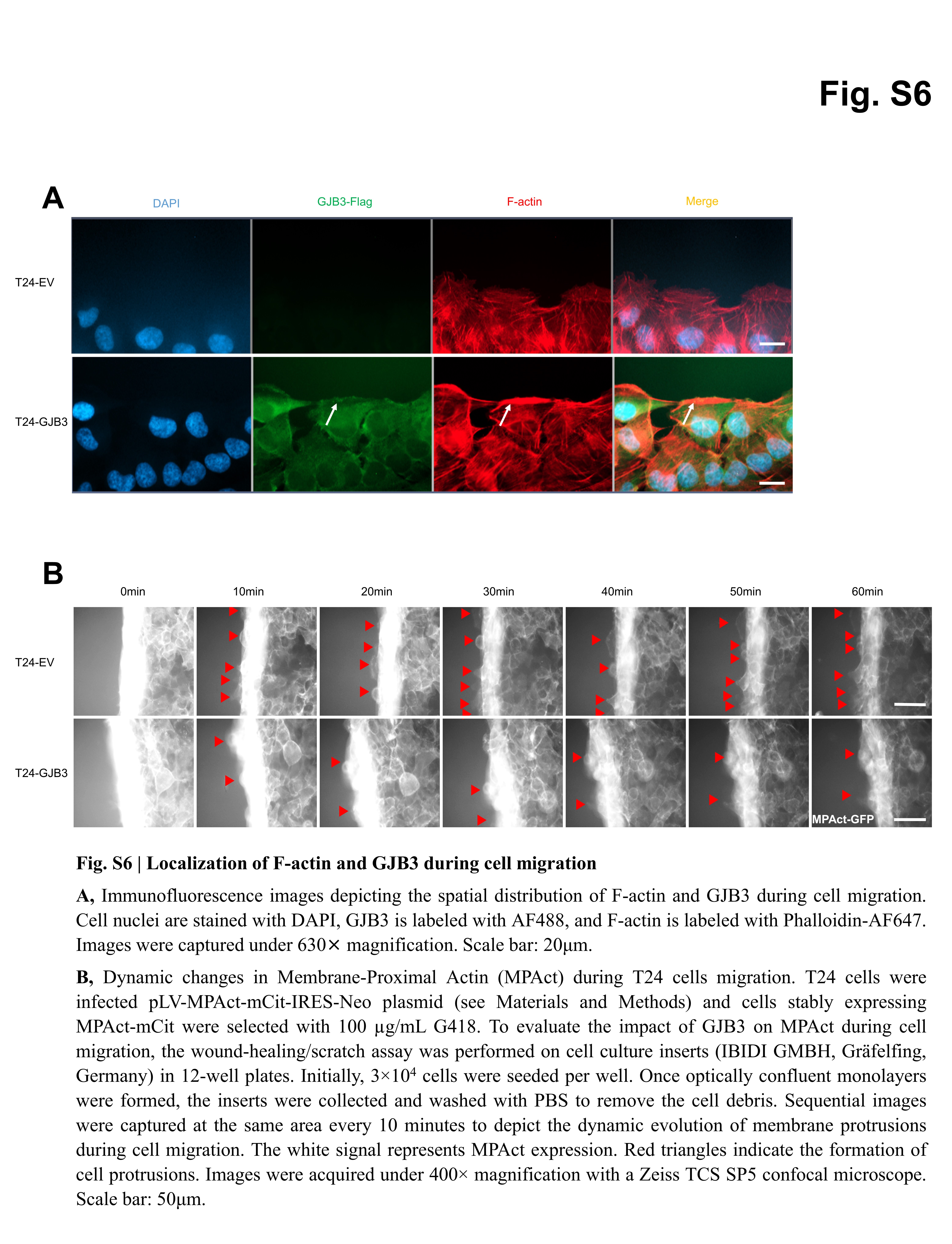

Supplement: Supplementary file 1 — Additional file 1: Supplementary figures and tables: Figure S1. Detection of GJB3 by IHC in human and mouse normal bladder tissues. Figure S2. Clinical data of patients with BC and the evaluation of patient survival. Figure S3. GJB3 has no impact on cell viability and proliferation. Figure S4. Example figure showing the measurement of the invasive capacity of cells in the ex vivo porcine bladder organ model. Figure S5. RT-qPCR for the detection mRNA levels of markers for epithelial-to-mesenchymal transition. Figure S6. Immunoflourescence on fixed cells and MPact live cell imaging for detecting potential colocalization of F-actin and GJB3 during cell migration. Table S1. Cloning primers. Table S2. ShRNA vectors and sequences. Table S3. Guide RNA vectors and sequences. Table S4. Primers for qPCR. [file 11658_2024_609_MOESM1_ESM.zip › Additional file 1/Fig. S6.JPG]

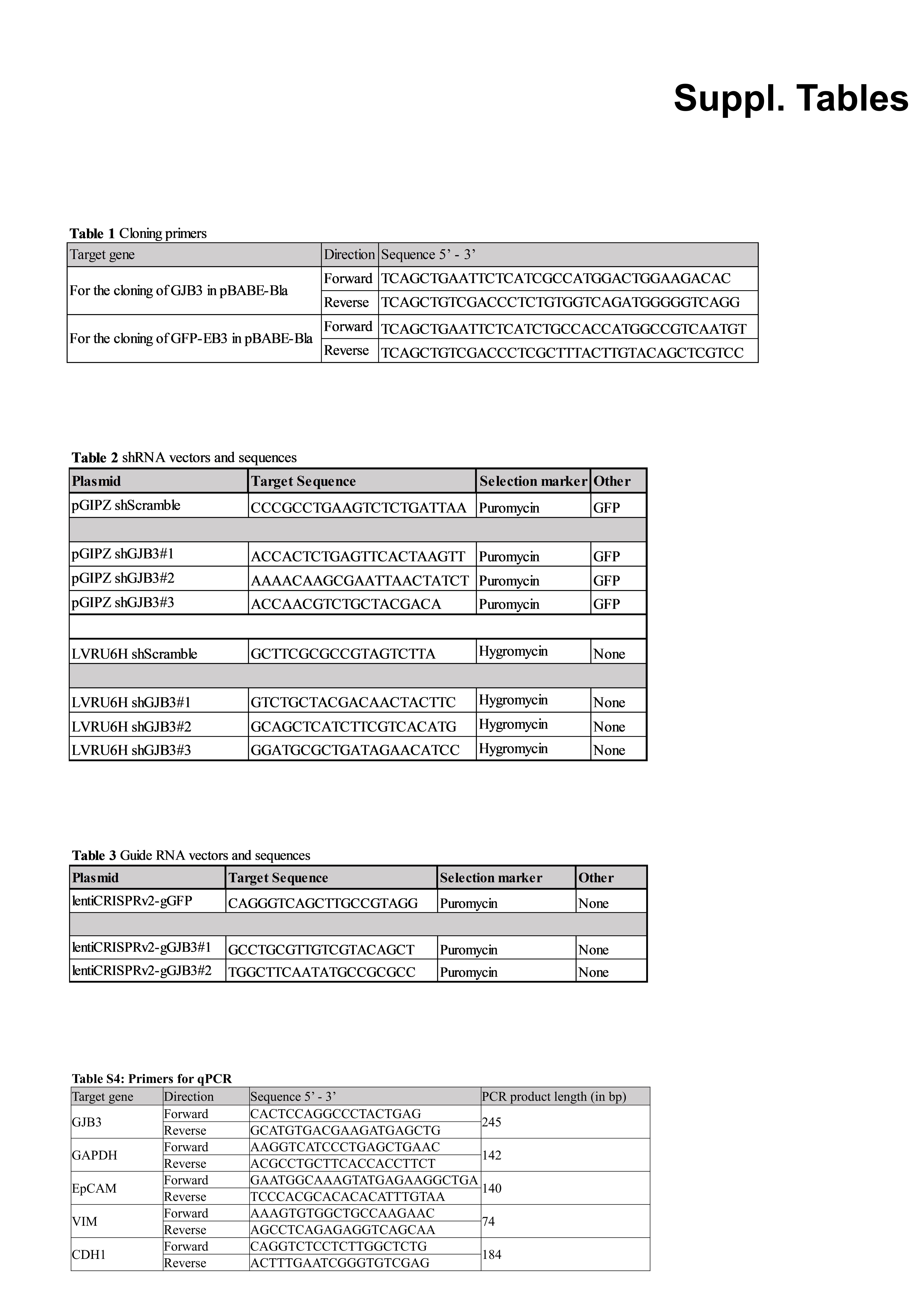

Supplement: Supplementary file 1 — Additional file 1: Supplementary figures and tables: Figure S1. Detection of GJB3 by IHC in human and mouse normal bladder tissues. Figure S2. Clinical data of patients with BC and the evaluation of patient survival. Figure S3. GJB3 has no impact on cell viability and proliferation. Figure S4. Example figure showing the measurement of the invasive capacity of cells in the ex vivo porcine bladder organ model. Figure S5. RT-qPCR for the detection mRNA levels of markers for epithelial-to-mesenchymal transition. Figure S6. Immunoflourescence on fixed cells and MPact live cell imaging for detecting potential colocalization of F-actin and GJB3 during cell migration. Table S1. Cloning primers. Table S2. ShRNA vectors and sequences. Table S3. Guide RNA vectors and sequences. Table S4. Primers for qPCR. [file 11658_2024_609_MOESM1_ESM.zip › Additional file 1/Suppl. Tables.JPG]
